# Promoter activity and transcriptome analyses decipher functions of *CgbHLH001* gene (*Chenopodium glaucum* L.) in response to abiotic stress

**DOI:** 10.1186/s12870-023-04128-8

**Published:** 2023-02-27

**Authors:** Zixin Zhou, Juan Wang, Qinghui Yu, Haiyan Lan

**Affiliations:** 1 Xinjiang Key Laboratory of Biological Resources and Genetic Engineering, College of Life Science and Technology, Xinjiang University, Urumqi, 830017 China; 2 Institute of Horticulture Crops, Xinjiang Academy of Agricultural Science, Urumqi, 830091 China

**Keywords:** *CgbHLH001*, *Chenopodium glaucum*, Promoter activity, Salt tolerance, Transcriptome analysis

## Abstract

**Background:**

Our previous studies revealed that CgbHLH001 transcription factor (TF) played an important role in abiotic stress tolerance, suggesting that its promoter was a potential target in response to stress signals. In addition, the regulatory mechanism of CgbHLH001 TF is still limited.

**Results:**

In the present study, a 1512 bp of 5’-flanking sequence of *CgbHLH001* gene was identified, and the sequence carried quite a few of *cis*-acting elements. The gene promoter displayed strong activity and was induced by multiple abiotic stress. A series of 5’-deletions of the promoter sequence resulted in a gradual decrease in its activity, especially, the 5’ untranslated region (UTR) was necessary to drive promoter activity. Further, *CgbHLH001* promoter drove its own gene overexpression ectopically at the transcriptional and translational levels, which in turn conferred the stress tolerance to transgenic *Arabidopsis*. Transcriptome analysis showed that salt stress induced a large number of genes involved in multiple biological regulatory processes. Differentially expressed genes (DEGs) that mediate phytohormone signal transduction and mitogen-activated protein kinase (MAPK) signaling pathway were widely induced and mostly upregulated under salt stress, and the transcription levels in *P*_*bHLH*_*::bHLH*-overexpressing transgenic lines were higher than that of *35S::bHLH* overexpression.

**Conclusions:**

The *CgbHLH001* promoter exhibited a positive response to abiotic stress and its 5’ UTR sequence enhanced the regulation of gene expression to stress. A few important pathways and putative key genes involved in salt tolerance were identified, which can be used to elucidate the mechanism of salt tolerance and decipher the regulatory mechanism of promoters to develop an adaptation strategy for desert halophytes.

**Supplementary Information:**

The online version contains supplementary material available at 10.1186/s12870-023-04128-8.

## Background

Gene expression is generally regulated at transcriptional and post-translational levels [1], in which the promoter plays an important role at the transcriptional level [2]. Various *cis*-acting elements are distributed at a high frequency throughout the upstream sequence around 1–3 kb region from the start codon ATG of a gene [3], including elements responsive to light, abiotic stress, hormone, and other stimuli, which provide the sites for transcription factors (TFs) [4]. Among promoter sequences, the 5’ UTR is vital for the control of gene expression. It may act as a translational enhancer or repressor during the initiation process [5, 6]. For example, the 5’ UTR of the soybean cytosolic glutamine synthetase β1 gene (*GS1*) enhances *GUS* expression for 20-fold at translational level [7]. Further studies indicate that the secondary structure, upstream start codon (uAUG), and upstream open reading frames (uORF) apply the vital function in the regulation of translational efficiency [8, 9]. Generally, the function of *cis*-acting elements can be dissected via truncations of the promoter sequence at the 5’ end. In plants, the recombinant *Promoter::GUS* (β-glucuronidase gene) construct and the *Agrobacterium*-mediated transient expression system, as well as the generation of transgenic plants, are commonly used to analyze promoter activity [10]. For example, the tomato *ELIP* (early light-inducible protein) gene promoter is identified with different positive or negative regulatory elements using seven 5’-deletion variants [11]. The understanding of promoter activity and the role of key elements is essential for utilizing new transgenic technologies in plant molecular breeding.

RNA-seq technology has been widely used to comparatively analyze changes in gene profile induced by stress and further explore the stress-responsive mechanisms. Currently, several plant species have been successfully investigated of the gene expression and function via transcriptomic analyses, such as *Arabidopsis thaliana* [12, 13], rice [14, 15], tobacco [16], maize [17], and cotton [18]. Plant species (*e.g.*, some halophytes) exhibited poor genetic transformation efficiency need to employ the transgenic *Arabidopsis* to explore gene functions. Overexpression of *Halostachys capsica HcSCL13* gene in transgenic *Arabidopsis* enhanced plant growth and salt tolerance, transcriptomic analysis revealed that various signaling pathways are involved in controlling plant development in *HcSCL13* transgenic *Arabidopsis* under salt stress [19]. Therefore, transcriptome analysis has become a powerful tool to decipher the molecular basis of plant in response to abiotic stress.

*Chenopodium glaucum*, as a pioneer halophyte distributed in semi-arid areas of the Xinjiang Uygur Autonomous Region in China, exhibits outstanding characteristics in response to abiotic stress [20]. Halophytes, such as *Salsola ferganica* and *Suaeda aralocaspica*, have generally evolved specified morphology or accessory structures (such as succulent leaves, trichomes, and heteromorphic seeds) to adapt to saline conditions, while *C. glaucum* exhibits normal plant morphology and leaf structures, but still can confer positive environmental impact in improving soil salinity [21, 22]. Therefore, elucidating the mechanism of stress tolerance in *C. glaucum* has a great significance. In our previous work, a bHLH transcription factor (TF) gene from *C. glaucum* (named as *CgbHLH001*) was characterized, which is important in stress response, especially drought tolerance [23], however, the regulatory mechanism of CgbHLH001 TF is still not clear. Based on its promoter sequence (with a potential target in response to stress signals), as well as the downstream gene network regulated by CgbHLH001 TF, in the present study, we further investigated the promoter activity and the mechanism of *CgbHLH001* gene driven by self-promoter in response to abiotic stress, and the regulatory networks and key genes responded to salt stress in *CgbHLH001* overexpressing *Arabidopsis* were also analyzed. Figuring out these details, we may gain further insight into the regulatory mechanism of bHLH TF in response to stress.

## Results

### Analysis of *CgbHLH001* promoter activity and 5’ UTR function

#### Analysis of the promoter sequence and expression pattern of *CgbHLH001* gene

**Fig. 1:**
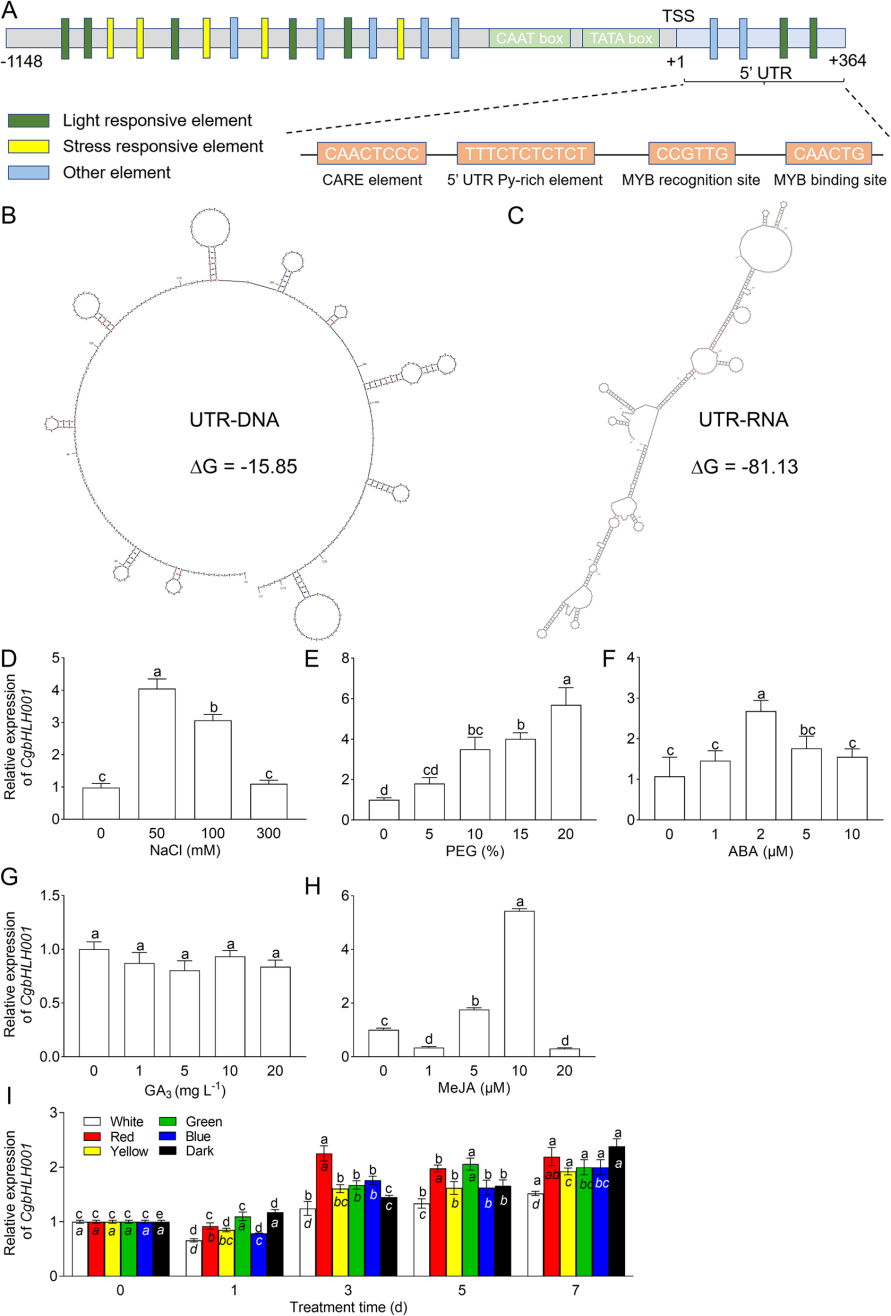
Analysis of the promoter and expression patterns of *CgbHLH001* gene. **A** Schematic diagram of the distribution of predicted *cis*-regulatory elements on *CgbHLH001* promoter. **B-C** Predicted secondary structures of DNA and RNA of the 5’ UTR. **D-I** Expression patterns of *CgbHLH001* in response to different treatments in *C. glaucum*. The wavelength of the different color light is: white (380–750 nm), red (621–750 nm), yellow (570–750 nm), green (491–570 nm), blue (450–490 nm). Different lowercase letters indicate the significant differences (*P* < 0.05) existing among different concentrations in **D**-**H**; in **I**, different standard lowercase letters indicate the significant differences among different treatment day(s) with the same light quality; different italic lowercase letters indicate the significant differences among different light qualities at the same treatment time. Values are means ± SD of three biological replicates with two technical replicates of each

By genomic walking technique, a 1512 bp of 5’ upstream sequence of *CgbHLH001* gene (GeneBank accession No. MW544164) was obtained, the transcription start site (TSS) was localized by TSSP software and assigned as ‘ + 1’ (Fig. 1A). A possible promoter sequence was predicted upstream between -1 bp and -1148 bp region. There were many putative *cis*-acting elements within this region, besides TATA box and CAAT box (core elements), elements on stress responsiveness, plant hormone regulation, light responsiveness, and TF-binding sites were found throughout the predicted promoter sequence, the detailed information was present in Table S1 of the Additional file 8. Notably, within the 5’ UTR region (a 364 bp sequence between the TSS and the ATG start codon), the typical Myb recognition and binding sites, and a Py-rich stretch (highly efficient transcription element) were found (Fig. 1A). The secondary structures of the 5’ UTR suggest a multiple stem-loop structure with -15.85 kcal mol^−1^ of the folding free energy (∆G) value in DNA (Fig. 1B) and -81.13 kcal mol^−1^ of ∆G value in RNA (Fig. 1C), these data imply that 5’ UTR sequence of *CgbHLH001* may play an important role in gene expression.

The transcriptional expression pattern of *CgbHLH001* gene was detected in *C. glaucum* before its promoter activity could be further explored. Our results showed that low salinity (50 and 100 mM NaCl) stimulated the accumulation of *CgbHLH001* transcripts (Fig. 1D); PEG increased gene expression level gradually and significantly within the test concentrations (0 to 20%) (Fig. 1E). For hormone treatments, ABA and MeJA enhanced the expression level at lower concentrations (the former at 2 μM, the latter at 5 and 10 μM), while GA_3_ had no effect on *CgbHLH001* expression at the test concentrations (Fig. 1F, G, H). When treated with different light qualities (including white, red, yellow, green, blue, and darkness), the expression level of *CgbHLH001* increased with the time extending, especially under red light and the darkness (Fig. 1I). Our results suggest that *cis*-elements in *CgbHLH001* promoter can respond to different stimuli and consequently activate the transcriptional expression of *CgbHLH001* gene.

#### Effect of 5’-deletions on the activity of *CgbHLH001* promoter

**Fig. 2:**
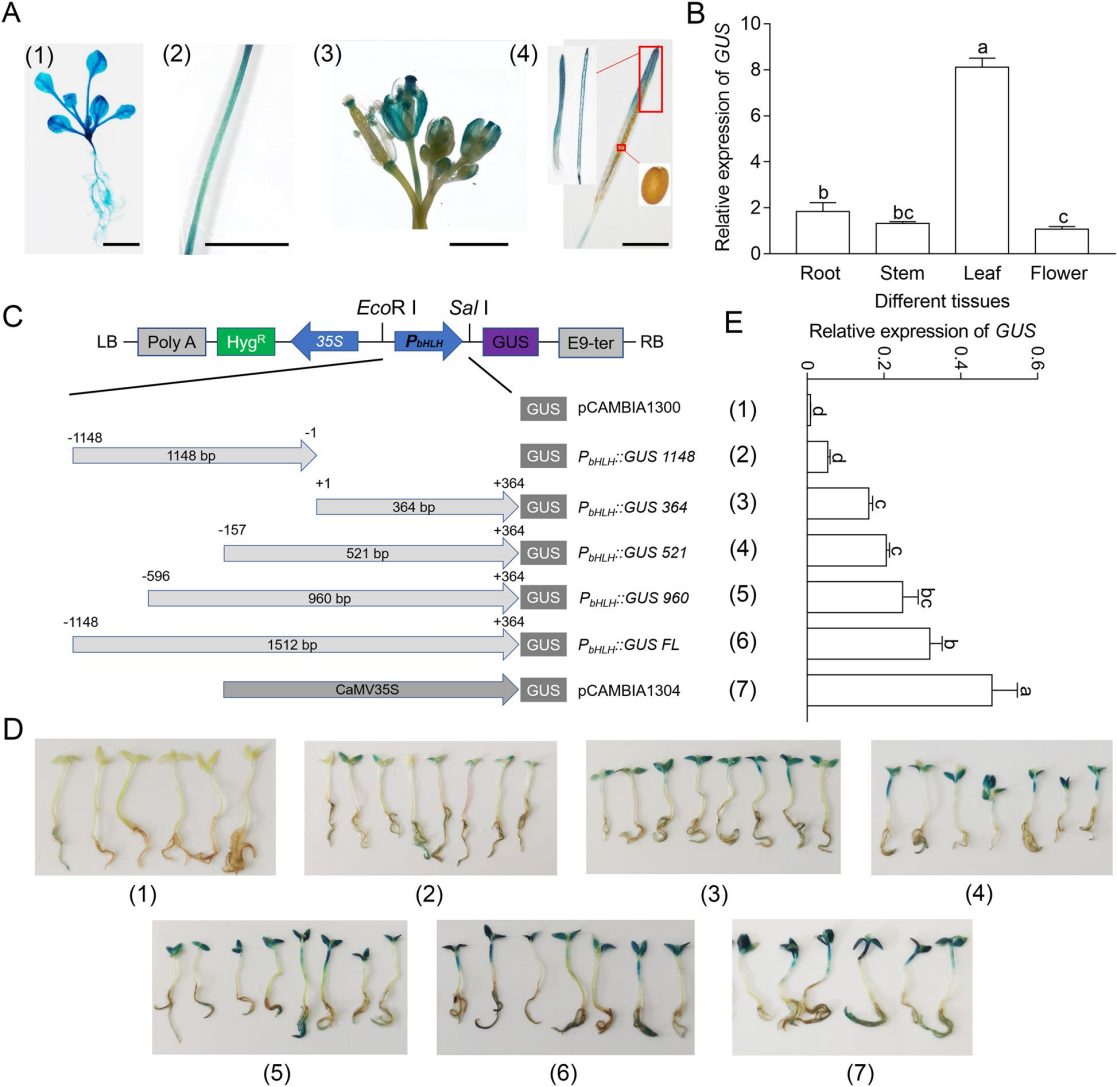
Analysis of *CgbHLH001* promoter activity by 5’-deletions. **A** Histochemical staining of *Arabidopsis* seedlings overexpressing *P*_*bHLH*_*::GUS FL* (full length of the *CgbHLH001* promoter)*.* (1) seedling; (2) stem; (3) flowers and early siliques; (4) mature silique, red rectangle on the silique is corresponding to the enlarged inset of seed (right) or pseudoseptum and pericarp (left). Scale bar = 0.5 cm. **B** Transcriptional expression of *GUS* gene in different tissues of *Arabidopsis* overexpressing *P*_*bHLH*_*::GUS FL*. **C** Schematic diagram of 5’-deletions of the promoter and constructs. Different truncated fragments of the promoter were constructed into plant expression vector pCAMBIA1300. (1) pCAMBIA1300: no promoter driving *GUS* gene; (2) *P*_*bHLH*_*::GUS 1148*, (3) *P*_*bHLH*_*::GUS 364*, (4) *P*_*bHLH*_*::GUS 521*, (5) *P*_*bHLH*_*::GUS 960*: different truncated fragments of *CgbHLH001* promoter driving *GUS* gene; (6) *P*_*bHLH*_::*GUS FL*: full length of the *CgbHLH001* promoter driving *GUS* gene; (7) pCAMBIA1304: *CaMV35S* promoter driving *GUS* gene. Hyg^R^: hygromycin resistant gene; E9-ter: terminator signal of the pea *rbcS-E9* gene. **D** GUS histochemical staining in *C. glaucum* seedlings transiently transformed with different truncations of the *CgbHLH001* promoter. **E** Transcriptional expression of *GUS* gene in accordance with treatment in **D**. Different lowercase letters indicate significant differences (*P* < 0.05) existing among different promoter truncations. In **D**, **E**, the number order is corresponding to the constructs in **C**

Transgenic *Arabidopsis* harboring *P*_*bHLH*_*::GUS* was generated to analyze the promoter activity. Histochemical staining showed that stronger blue color was present in leaves compared with that of roots, stems or flowers; shoot apical meristem was also observed apparently blue color, the pseudoseptum and pericarps of the mature silique presented less blue color, and the seed displayed no visible GUS staining (Fig. 2A). Correspondingly, the transcript accumulation of *GUS* gene in different tissues showed the similar tendency, the leaves exhibited the highest while flowers had the lowest expression level (Fig. 2B). A series of truncates of the *CgbHLH001* promoter were generated and fused to *GUS* reporter gene to define the regions of importance on promoter activity (Fig. 2C), then analyzed by transient expression system in *C. glaucum* seedlings. With the 5’-deletion increasing, the GUS expression decreased significantly in both mRNA (relative expression) and protein (enzyme activity) levels (Fig. 2D, E). Interestingly, most of the promoter activity was lost after the 5’ UTR-deletion (*P*_*bHLH*_*::GUS 1148*), suggesting that the 5’ UTR sequence plays an important effect on promoter activity of *CgbHLH001*.

#### Effects of abiotic stress on the activity of *CgbHLH001 *promoter in transgenic *Arabidopsis*

**Fig. 3:**
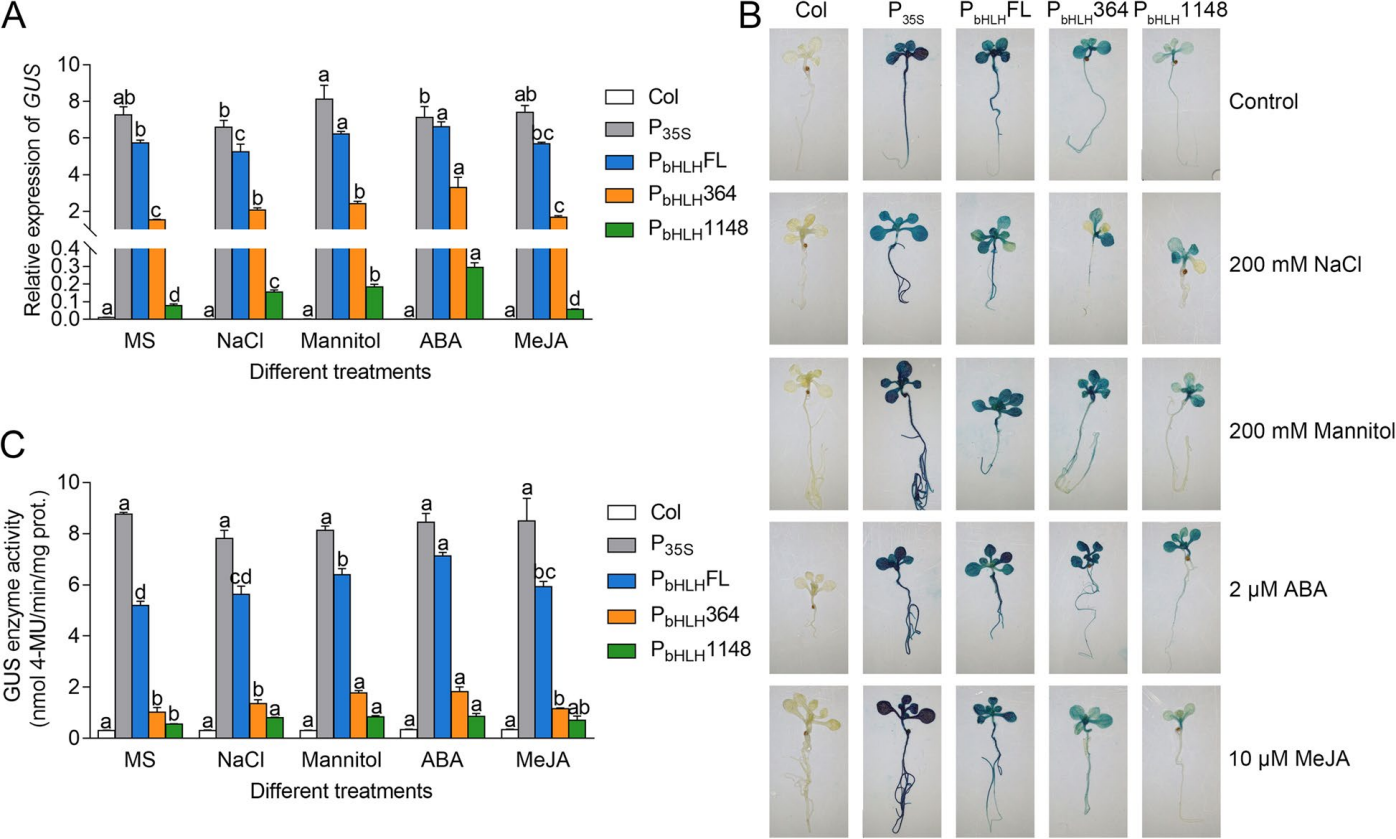
Analysis of *CgbHLH001* promoter activity under abiotic stress. **A** Relative expression of *GUS* gene driven by P_bHLH_FL, P_bHLH_364, and P_bHLH_1148. *CaMV35S* promoter (P_35S_) was used as the positive control; Col was used as the negative control. P_bHLH_FL: full-length *CgbHLH001* promoter driving *GUS* gene; P_bHLH_364: 5’ UTR sequence of *CgbHLH001* promoter driving *GUS* gene; P_bHLH_1148: 5’ UTR deletion of full-length *CgbHLH001* promoter driving *GUS* gene. **B** GUS staining of transgenic *Arabidopsis* seedlings. **C** Detection of GUS enzyme activity. The GUS activity was expressed as nmol 4-methylumbelliferone per minute per mg protein. Bars represent means ± SD of three biological replicates. Different lowercase letters indicate significant differences (*P* < 0.05) existing among different treatments in **A** and **C**

To further characterize the activity of *CgbHLH001* promoter in response to the abiotic stress, *GUS* expression and fluorometric GUS activity were analyzed in transgenic *Arabidopsis* overexpressing *CaMV35S*::*GUS*, *P*_*bHLH*_* FL*::*GUS*, *P*_*bHLH*_* 364*::*GUS*, and *P*_*bHLH*_ *1148::GUS*. Under normal condition, transgenic seedlings with *CaMV35S* promoter exhibited the highest *GUS* expression level, the full-length promoter ranked second, which also had relatively higher activity; the 5’ UTR sequence could drive *GUS* expression at certain level, however, the promoter without 5’ UTR sequence lost most of the activity (Fig. 3A). When subjected to abiotic stress or phytohormonal treatments, *GUS* gene driven by *P*_*bHLH*_* FL* was significantly promoted under mannitol and ABA treatments, those driven by *P*_*bHLH*_* 364* or *P*_*bHLH*_ 1148 were also significantly enhanced compared to control, except for MeJA treatment (Fig. 3A). Similarly, stronger GUS staining and higher GUS enzyme activity of *P*_*bHLH*_* FL* transgenic lines were observed under stress treatments, except for 200 mM NaCl treatment (which may partly be the leaf damage under stress); seedlings harboring with 5’ UTR of *CgbHLH001* promoter was activated by mannitol and ABA treatments, while *CaMV35S* promoter showed no significant difference in promotion of GUS activity under abiotic stress compared with that of normal condition (Fig. 3B, C). All these results indicate that *CgbHLH001* promoter can respond to abiotic stress and the 5’ UTR is necessary for promoter activity.

#### Responses of *CgbHLH001* gene to abiotic stress driven by self-promoter

**Fig. 4:**
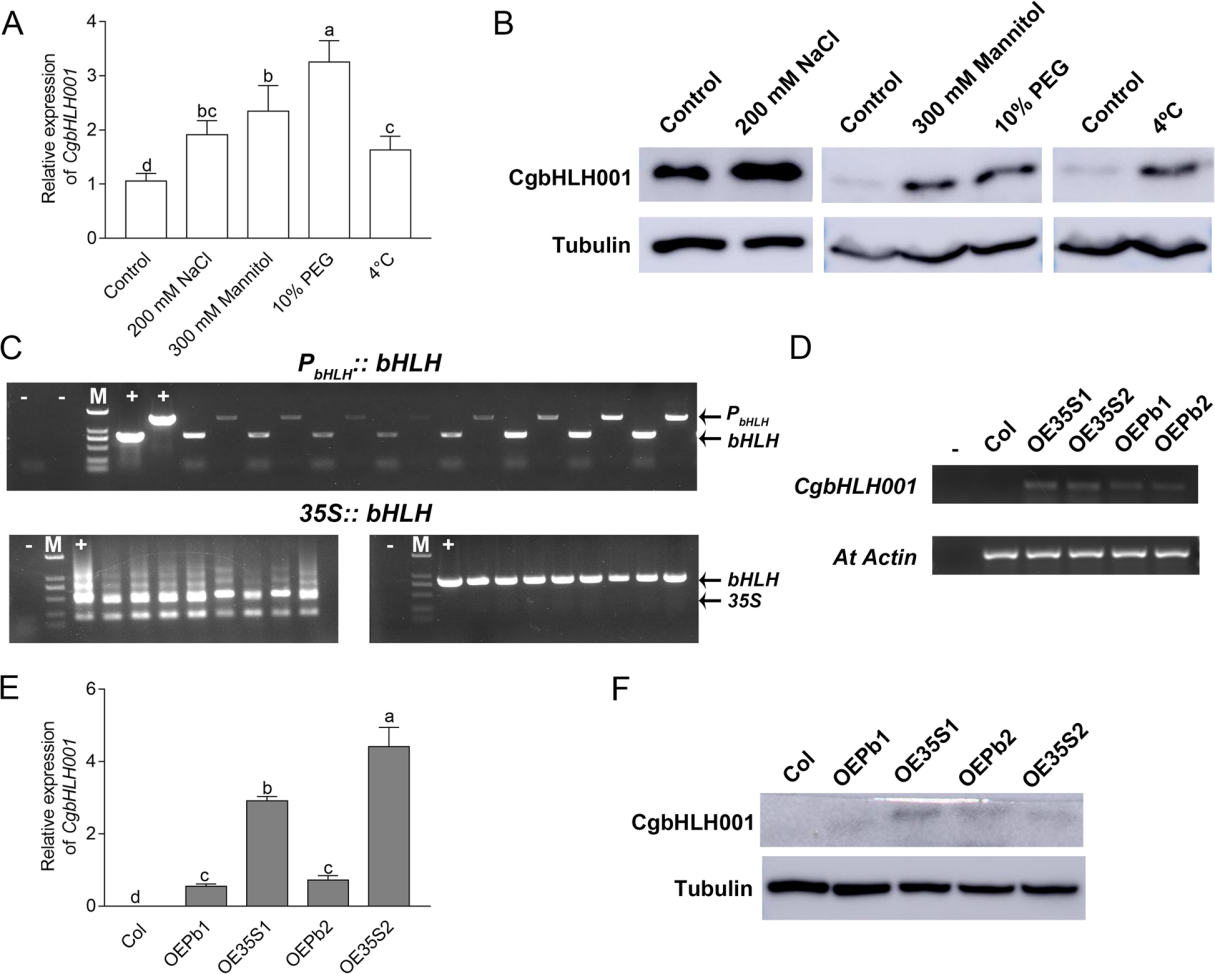
Analysis of *CgbHLH001* expression in *C. glaucum* and identification of transgenic *Arabidopsis* overexpressing *CgbHLH001*. **A-B** Detection of *CgbHLH001* expression in *C. glaucum* under different abiotic stress at mRNA and protein levels. **C-F** Identification of *CgbHLH001* in transgenic *Arabidopsis* by PCR, RT-PCR, qRT-PCR and Western blot. In **A**, different lowercase letters above the columns indicate significant differences (*P* < 0.05) between different treatments; in **C**, + : positive control; -: negative control; M: DL2000 DNA marker; in **D**, -: negative control; *Atactin* served as the internal reference; in **E**, different lowercase letters above the columns indicate significant differences (*P* < 0.05) between different *Arabidopsis* lines; in **F**, Tubulin served as the internal reference. Original images of gels and blots were present in Supporting Fig. 1 in the Additional file 14

The transcripts and protein of *CgbHLH001* in *C. glaucum* were significantly accumulated when subjected to different abiotic stress compared to that of normal condition (Fig. 4A, B), suggesting the important role of *CgbHLH001* gene in stress response. To characterize the function of *CgbHLH001* gene and the effect of its promoter in response to abiotic stress, T3 generation transgenic *Arabidopsis* lines overexpressing *P*_*bHLH*_*::bHLH* or *35S::bHLH* were identified by genomic PCR, semi-quantitative RT-PCR, quantitative real-time PCR (qRT-PCR) and Western blot (Fig. 4C-F). The expression of *CgbHLH001* gene driven by *CaMV35S* promoter was significantly higher than that of driven by self-promoter in both mRNA and protein levels (Fig. 4E, F).

**Fig. 5:**
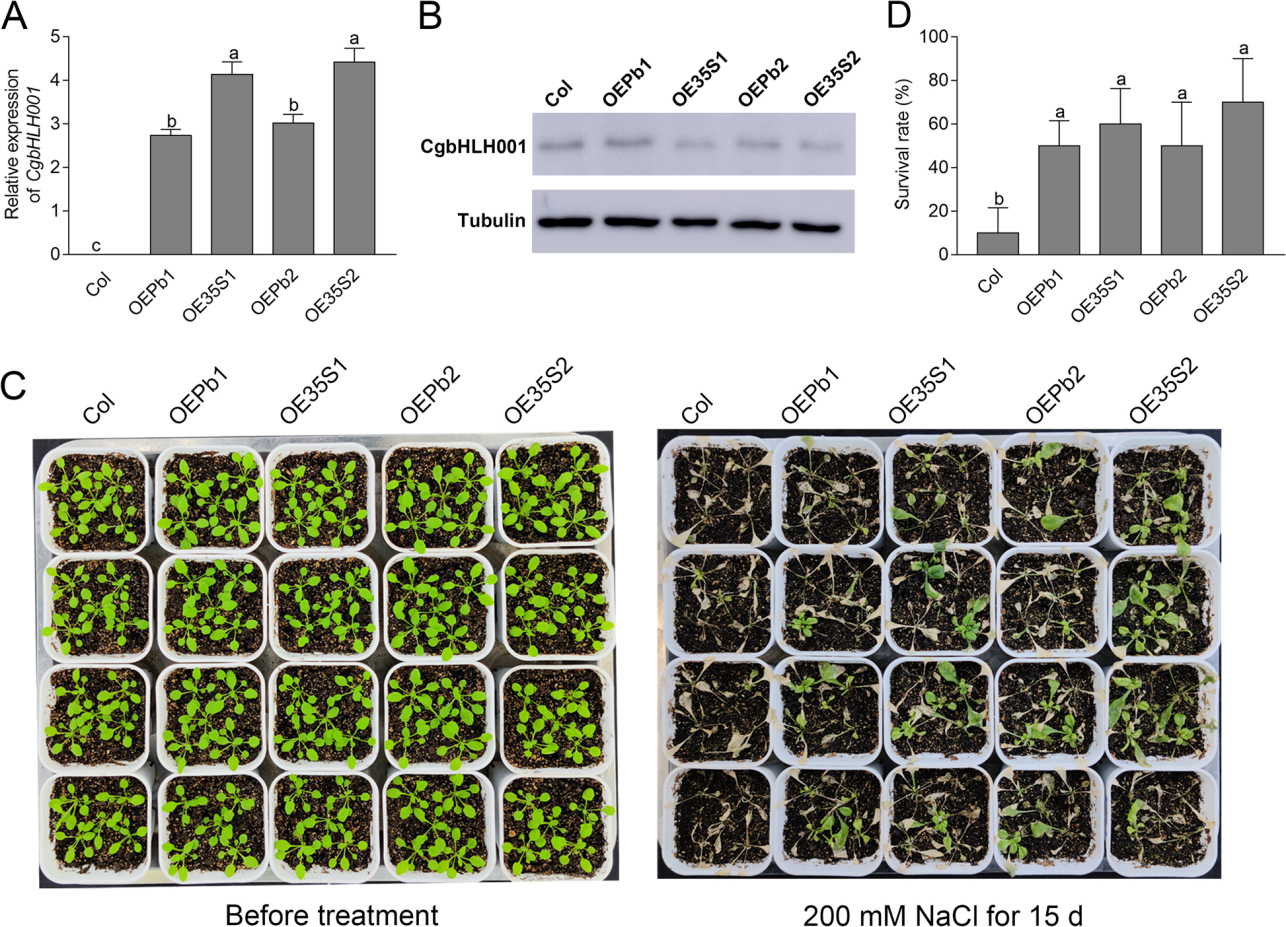
Phenotypic performance and gene expression of transgenic *Arabidopsis* lines (overexpressing *35S::bHLH* and *P*_*bHLH*_*::bHLH*) in response to 200 mM NaCl treatment. **A** Transcriptional expression of *CgbHLH001* gene. **B** Translational expression of CgbHLH001 protein. **C**-**D** Phenotypic observation and survival percentage of transgenic *Arabidopsis*. OE35S1, 2: *35S::bHLH*-overexpressing transgenic *Arabidopsis* line 1, 2; OEPb1, 2: *P*_*bHLH*_*::bHLH*-overexpressing transgenic *Arabidopsis* line 1, 2. Different lowercase letters in **A**, **D** indicate significant differences (*P* < 0.05) existing between different transgenic lines. Original images of blots were present in Supporting Fig. 1 in the Additional file 14

To further verify the combined effect of the promoter activity on *CgbHLH001* gene function, the phenotypic performances and gene expressions at transcriptional and translational levels were investigated by employing transgenic *Arabidopsis* lines overexpressing *P*_*bHLH*_*::bHLH* and *35S::bHLH*. The expression of *CgbHLH001* (mRNA and protein levels) was activated when exposed to salt stress, although *P*_*bHLH*_ promoter had less effect on gene expression compared with the *CaMV35S* promoter, and changes of protein level were not much more compared with transcripts (Fig. 5A, B). Moreover, WT suffered more than transgenic lines when subjected to 200 mM NaCl treatment for 15 d, in which transgenic *Arabidopsis* overexpressing *35S::bHLH* performed better than that of overexpressing *P*_*bHLH*_*::bHLH* (Fig. 5C, D). Further analyses of *CgbHLH001* in response to drought and cold treatments revealed that increased gene expression and alleviated damage were observed in transgenic lines, and the *35S::bHLH*-overexpressing lines had better performance (Fig. S1; Fig. S2 in the Additional files 1, 2). These results indicate that *CgbHLH001* promoter is stress-inducible and can drive downstream genes (including *CgbHLH001*) positively in response to abiotic stress.

### Analysis of transcriptomic data

#### Overview of the RNA-seq data

Based on the good performance of transgenic *Arabidopsis* overexpressing *CgbHLH001* in response to salt stress, we expect to further elucidate the regulatory mechanism of the gene. Transcriptomic data between WT and transgenic lines (*35S::bHLH* or *P*_*bHLH*_*::bHLH*) under normal condition or salt treatment for 1 h were analyzed. In total, 18 libraries were constructed and sequenced. For each sample, 92.39% to 95.71% reads were mapped to the *Arabidopsis* reference genome after filtering low-quality reads (Table S2 in the Additional file 9). The pairwise Pearson’s correlation coefficients indicated a high consistency among biological replicates (Fig. S3A in the Additional file 3). The principal component analysis (PCA) showed that the transgenic lines were distal to the WT, especially *P*_*bHLH*_*::bHLH*-overexpressing line under salt treatment (Fig. S3B in the Additional file 3), suggesting that transgenic *Arabidopsis* can obviously respond to stress. These results indicated that the RNA-seq data were reliable and could be used for further analysis.

#### Identification of DEGs

**Fig. 6:**
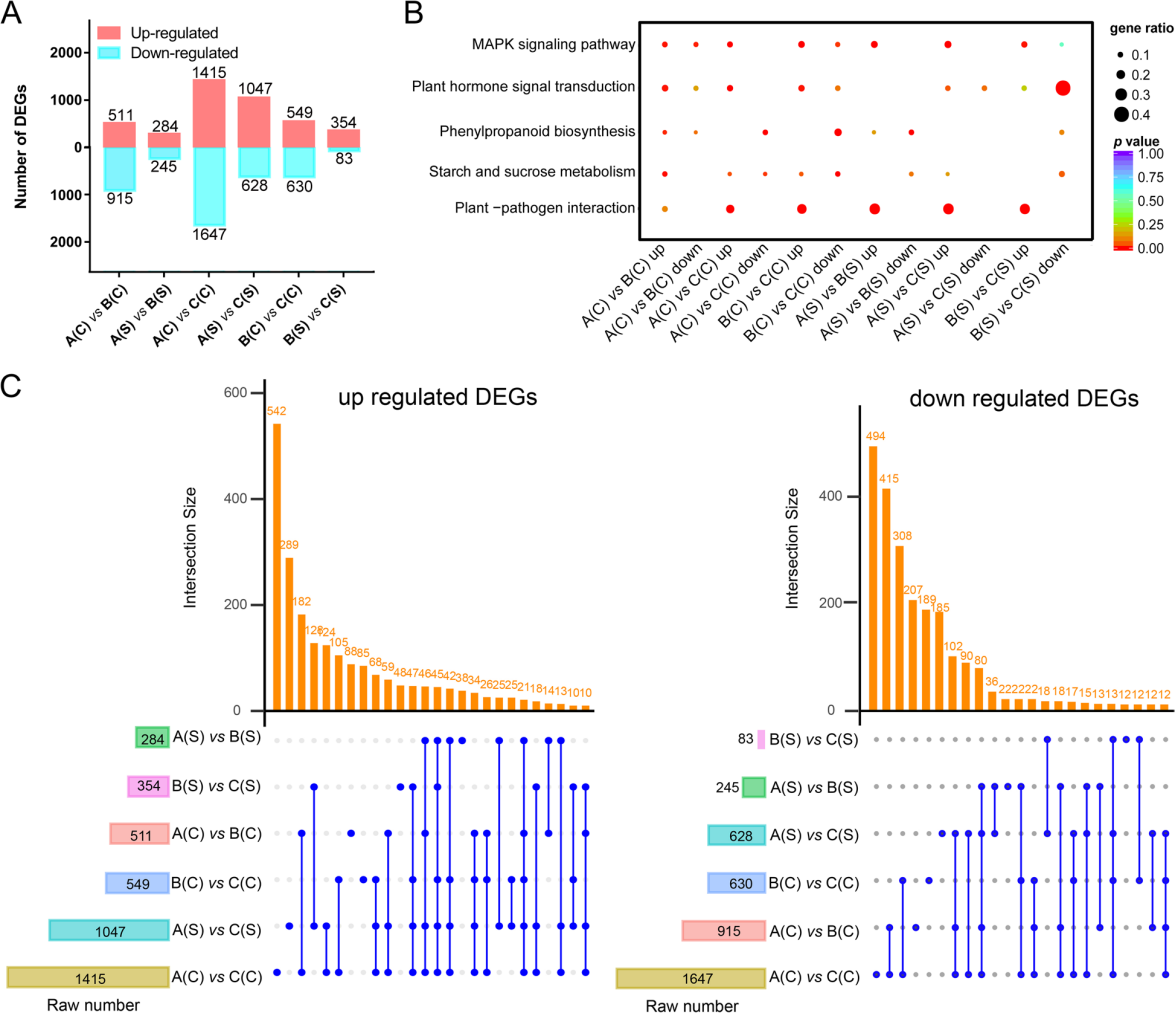
Numbers of DEGs identified in various comparisons. **A** Bar graph presents the number of up- and down-regulated DEGs in various comparisons. **B** KEGG pathway enrichment analysis on the data listed in **A**. **C** Upset graphs displaying shared and unique DEGs identified in various comparisons. A: wild type (Col-0); B: *35S::bHLH*-overexpressing transgenic *Arabidopsis*; C: *P*_*bHLH*_*::bHLH*-overexpressing transgenic *Arabidopsis*; (C): normal condition; (S): salt treatment

Pairwise comparisons were conducted among WT (A), *35S::bHLH* (B) and *P*_*bHLH*_*::bHLH* (C) under normal condition (C) or salt treatment (S) [A(C) *vs* B(C), A(S) *vs* B(S), A(C) *vs* C(C), A(S) *vs* C(S), B(C) *vs* C(C), and B(S) *vs* C(S)] to identify DEGs in response to abiotic stress. Of those, *35S::bHLH* and *P*_*bHLH*_*::bHLH* under salt condition [B(S) *vs* C(S)] exhibited the least differences with 354 upregulated and 83 downregulated DEGs, indicating that the two transgenic *Arabidopsis* exhibited similar response to salt stress. In contrast, WT and *P*_*bHLH*_*::bHLH* under normal condition [A(C) *vs* C(C)] exhibited the largest differences with 3062 DEGs (1415 upregulated and 1647 downregulated), implying significant difference at transcriptional level in *P*_*bHLH*_*::bHLH* transgenic *Arabidopsis*, which were much higher than that in *35S::bHLH* transgenic lines (Fig. 6A). KEGG analyses revealed that the most enriched pathways were related to “MAPK signaling pathway” “plant hormone signal transduction” “phenylpropanoid biosynthesis” “starch and sucrose metabolism” and “plant-pathogen interaction” (Fig. 6B).

The upregulated and downregulated DEGs among six comparisons were sorted out to analyze the transcriptional change under salt stress (Fig. 6C). The results revealed 542 DEGs exclusively upregulated and 494 DEGs exclusively downregulated in [A(C) *vs* C(C)], the former were enriched in photosynthesis (including photosystem I and II), while the latter were in response to hormones (such as cytokinin and gibberellin) (Fig. S4 A, B in the Additional file 4). However, 88 DEGs were exclusively upregulated in [A(C) *vs* B(C)] and enriched in plant developmental process; whereas 207 DEGs were exclusively downregulated in response to wounding and stress (Fig. S4 C, D in the Additional file 4). With the maximum number of overlapping DEGs between [A(C) *vs* C(C) and A(C) *vs* B(C)], 182 genes were commonly upregulated and 415 genes were downregulated. The most enriched GO terms were related to cellular components and plant growth and metabolism (Fig. S4 E, F in the Additional file 4).

### Characterization of the candidate genes and signaling pathways regulated by *CgbHLH001* gene and its promoter

#### Comparing between *35S::bHLH* and *P*_*bHLH*_*::bHLH* transgenic *Arabidopsis* to characterize the function of *CgbHLH001* promoter

**Fig. 7:**
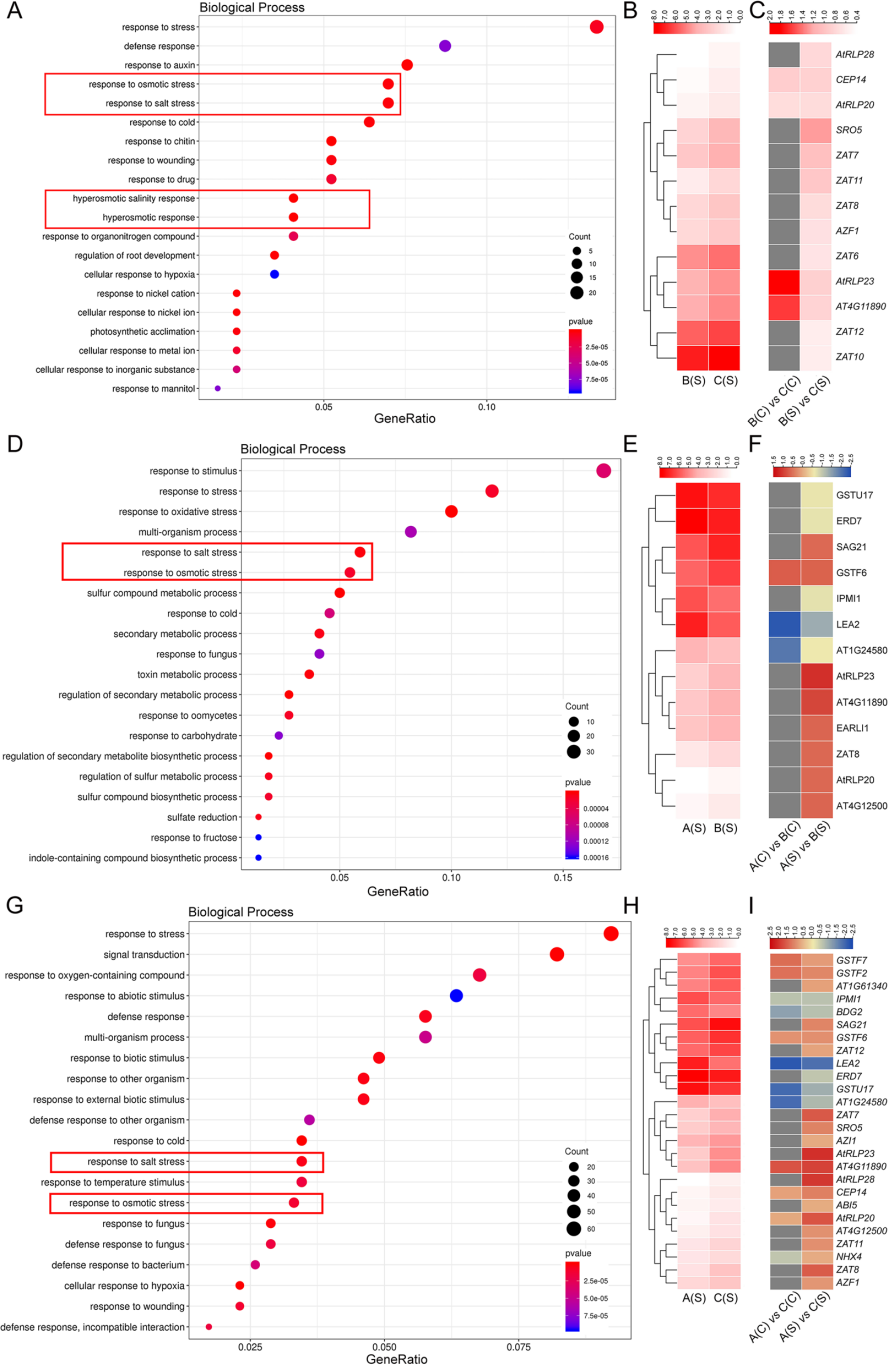
Characterization of salt stress-related genes induced by *CgbHLH001* gene and its promoter. **A-C** Comparison of B(S) *vs* C(S); **D-F** Comparison of A(S) *vs* B(S); **G-I** Comparison of A(S) *vs* C(S). **A**, **D**, **G** GO annotation of DEGs in biological process. **B**, **C**, **E**, **F**, **H**, **I** Heatmaps of selected DEGs related to salt stress based on FPKM values (**B**, **E**, **H**) or log_2_FC values (**C**, **F**, **I**). In (**A**, **D**, **G**), gene ratio represents the percentage of selected genes, the circle size represents gene numbers, the bigger the circle, the more the gene numbers. The color of circle represents the *p* value, the darker the color, the smaller the *p* value with higher significant difference. In (**B**, **E**, **H**), the FPKM values of DEGs were standardized by log_2_ (FPKM + 1). The darker the color, the higher the gene expression level. The left in (**B**, **E**, **H**) was clustered by gene expression level. The right in (**C**, **F**, **I**) represent gene name, which is consistent with that in (**B**, **E**, **H**). The darker the color, the larger the gene fold change. A: wild type (Col-0); B: *35S::bHLH*-overexpressing transgenic *Arabidopsis*; C: *P*_*bHLH*_*::bHLH*-overexpressing transgenic *Arabidopsis*; (C): normal condition; (S): salt treatment

Overexpressing *35S::bHLH* and *P*_*bHLH*_*::bHLH* transgenic *Arabidopsis* under salt stress [B(S) *vs* C(S)] were selected for analysis. GO annotation in biological process showed that DEGs were involved in plant development (auxin, chitin, root development, and photosynthetic acclimation) and stress response (defense, osmotic, salt, cold, wounding, and hyperosmotic salinity) (Fig. 7A). Furthermore, terms related to salt and osmotic stress were selected to narrow down the candidate genes. All of the 13 DEGs identified were upregulated: 4 genes were commonly upregulated in [B(C) *vs* C(C) and B(S) *vs* C(S)], the rest were exclusively upregulated in [B(S) *vs* C(S)] (Fig. 7B, C). Interestingly, 6 DEGs were identified belonging to ZAT protein family (C2H2 zinc fingers superfamily), which regulate plant development and respond to diverse stress [24]. In addition, the top 50 DEGs with the highest fold change (FC) were selected for functional annotation. The most enriched GO terms were involved in defense response, photosynthetic acclimation, stress response and ROS metabolic process (Fig. S5A in the Additional file 5). All the results showed that *CgbHLH001* promoter could activate the downstream stress-responsive genes which consequently mediate the stress tolerance.

#### The regulation of *CgbHLH001* gene overexpression in *Arabidopsis*

Our results showed that overexpressing *CgbHLH001* gene enhanced the abiotic stress tolerance of transgenic *Arabidopsis*. Here, the WT and *35S::bHLH* transgenic lines under salt stress [A(S) *vs* B(S)] were compared for further analyses. GO annotation associated with biological process revealed that salt/osmotic stress-related DEGs were enriched, a total of 13 DEGs (5 downregulated, 8 upregulated) were screened, which included an upregulated gene *SAG21* (senescence-associated gene 21) with higher expression profile (Fig. 7D, E). Three genes were commonly expressed in both [A(C) *vs* B(C) and A(S) *vs* B(S)], two of which were downregulated, while the expression was improved under salt treatment (Fig. 7F). Similarly, the most enriched GO terms of the top 50 DEGs with the highest FC were related to kinase activity, signal transduction, secondary metabolic process and response to oxidative stress, indicating that the overexpressing *CgbHLH001* gene may mediate signal transduction and hormone-related pathway in response to external stress (Fig. S5B in the Additional file 5).

#### The response of *CgbHLH001* gene overexpression induced by its promoter to salt stress

**Fig. 8:**
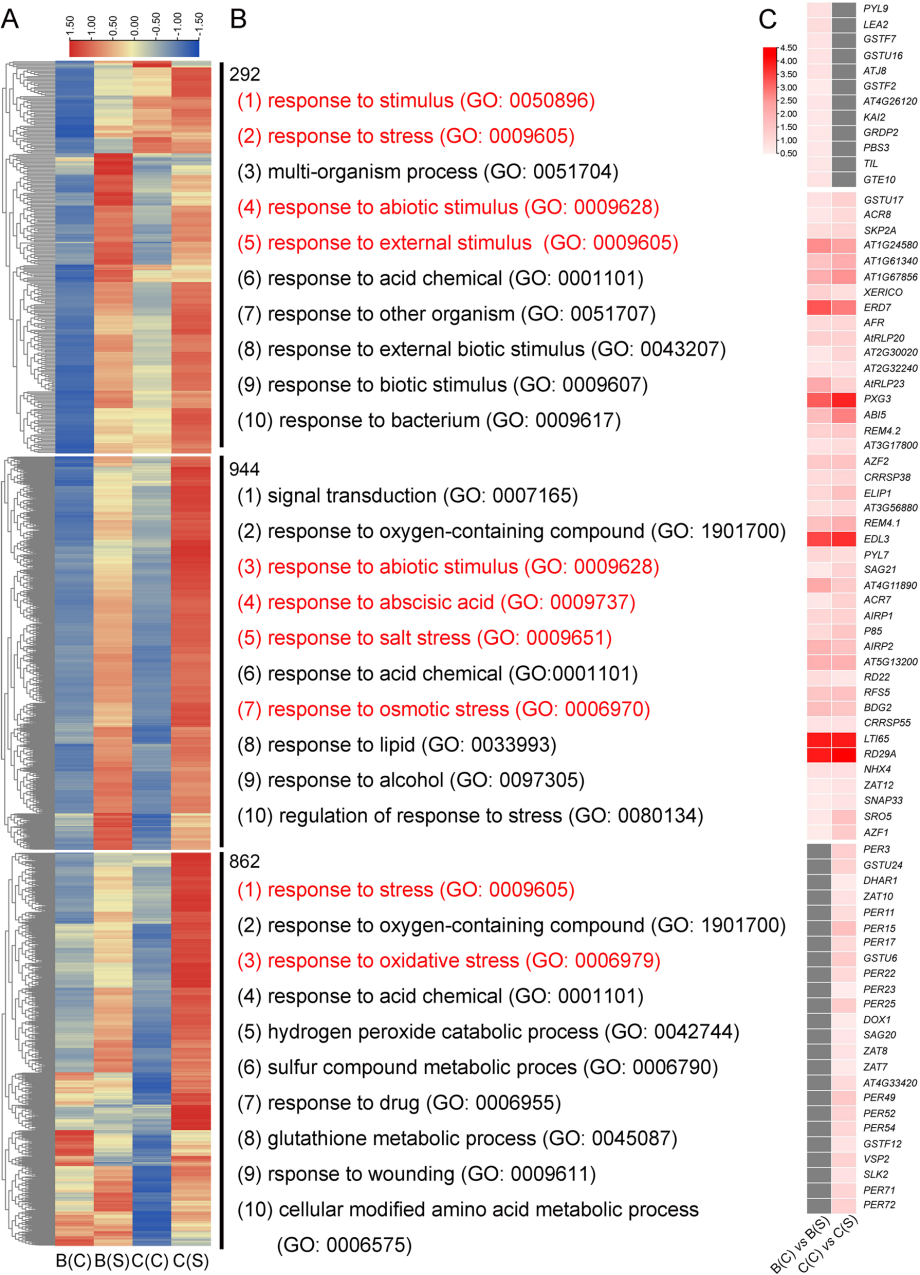
Characterization of upregulated DEGs in transgenic *Arabidopsis* under salt stress. **A** The heatmaps of DEGs between B(C) *vs* B(S) and C(C) *vs* C(S). **B** Top 10 GO functional annotations in biological process corresponding to **A**. **C** Heatmaps of DEGs related to salt stress based on log_2_FC values. In **A**, the heatmap was generated based on the FPKM value (Z-score method). In **B**, the red font indicated GO terms related to abiotic stress. B: *35S::bHLH*-overexpressing transgenic *Arabidopsis*; C: *P*_*bHLH*_*::bHLH*-overexpressing transgenic *Arabidopsis*; (C): normal condition; (S): salt treatment

The phenotypic observation revealed an improved performance of transgenic *Arabidopsis* than WT in plant growth and development following salt treatments. DEGs of transgenic *Arabidopsis* before and after salt stress [B(C) *vs* B(S) and C(C) *vs* C(S)] were identified to elucidate the mechanism of *CgbHLH001* overexpression in response to salt stress. The number of DEGs in *P*_*bHLH*_*::bHLH* transgenic lines [C(C) *vs* C(S)] was the highest (Fig. S6A in the Additional file 6). The Venn diagrams were generated to illustrate the similarities and differences between upregulated and downregulated genes (Fig. S6B, C in the Additional file 6). Furthermore, analysis of GO enrichment showed that the upregulated DEGs were positively correlated with multiple abiotic stress responses (Fig. S6D, E in the Additional file 6). Therefore, all the upregulated genes were selected to elucidate the salt-responsive mechanism. A total of 2098 upregulated DEGs were divided into three groups, including DEGs exclusively upregulated in [B(C) *vs* B(S)] (292), [C(C) *vs* C(S)] (862) or DEGs commonly upregulated in [B(C) *vs* B(S) and C(C) *vs* C(S)] (944) (Fig. 8A). GO analyses in biological process showed that all the three groups were involved in abiotic stress responses, especially the commonly upregulated DEGs, which covered more stress-related genes and presented higher expression levels after salt treatment, such as *ERD7* (early-responsive to dehydration 7), *EDL3* (EID1-like F-box protein 3), *LTI65* (low-temperature-induced 65 kDa protein), and *RD29A* (desiccation-responsive protein 29A) (Fig. 8B, C). The top 30 DEGs identified by FC values in [B(C) *vs* B(S) and C(C) *vs* C(S)] included a proportion of TFs and stress-related genes (Table S3 in the Additional file 10). In this study, a number of differentially expressed (DE) TFs were identified to understand the contribution of TFs in response to salt stress, including AP2/ERF-ERF, bHLH, MYB, WRKY, NAC, and bZIP (Table S4 in the Additional file 11), which plays important roles during stress responses in plants. Among these, C2C2-GATA, TCP and B3 TFs were exclusively downregulated, while C3H was exclusively upregulated. The number of upregulated genes in AP2/ERF-ERF, MYB, NAC, WRKY was significantly higher than the downregulated genes. The better performance of transgenic *Arabidopsis* under abiotic stress should be attributed to the significant expression of all the stress-related genes, especially the commonly upregulated DEGs.

Furthermore, we compared WT and *P*_*bHLH*_*::bHLH* transgenic *Arabidopsis* under salt treatment [A(S) *vs* C(S)] to explore the regulation of *CgbHLH001* gene driven by self-promoter. GO enrichment in biological process showed that stress-related terms were widely enriched, including response to abiotic stimuli (cold, salt, temperature, and osmotic stress) and biotic stimuli (defense, wounding, fungus and bacterium) (Fig. 7G). Accordingly, 27 DEGs related to salt and osmotic stress were identified, among them, 21 genes were upregulated and 6 genes were downregulated (Fig. 7H). Twelve DEGs were commonly expressed in both [A(C) *vs* C(C)] and [A(S) *vs* C(S)], especially the *NHX3* (Na^+^/H^+^ antiporter), which was downregulated under normal condition while upregulated after salt treatment. The remained 15 genes were exclusively expressed in [A(S) *vs* C(S)], all of them were upregulated (Fig. 7I). In addition, the top 50 DEGs of the most enriched GO terms identified by FC values were widely related to responses of abiotic stress (mannitol, salt) and biotic stimuli (Fig. S5C in the Additional file 5). All the results showed that overexpressing *CgbHLH001* gene driven by self-promoter presented more positive response to abiotic stress.

#### Gene expression patterns and functional enrichment of DEGs

**Fig. 9:**
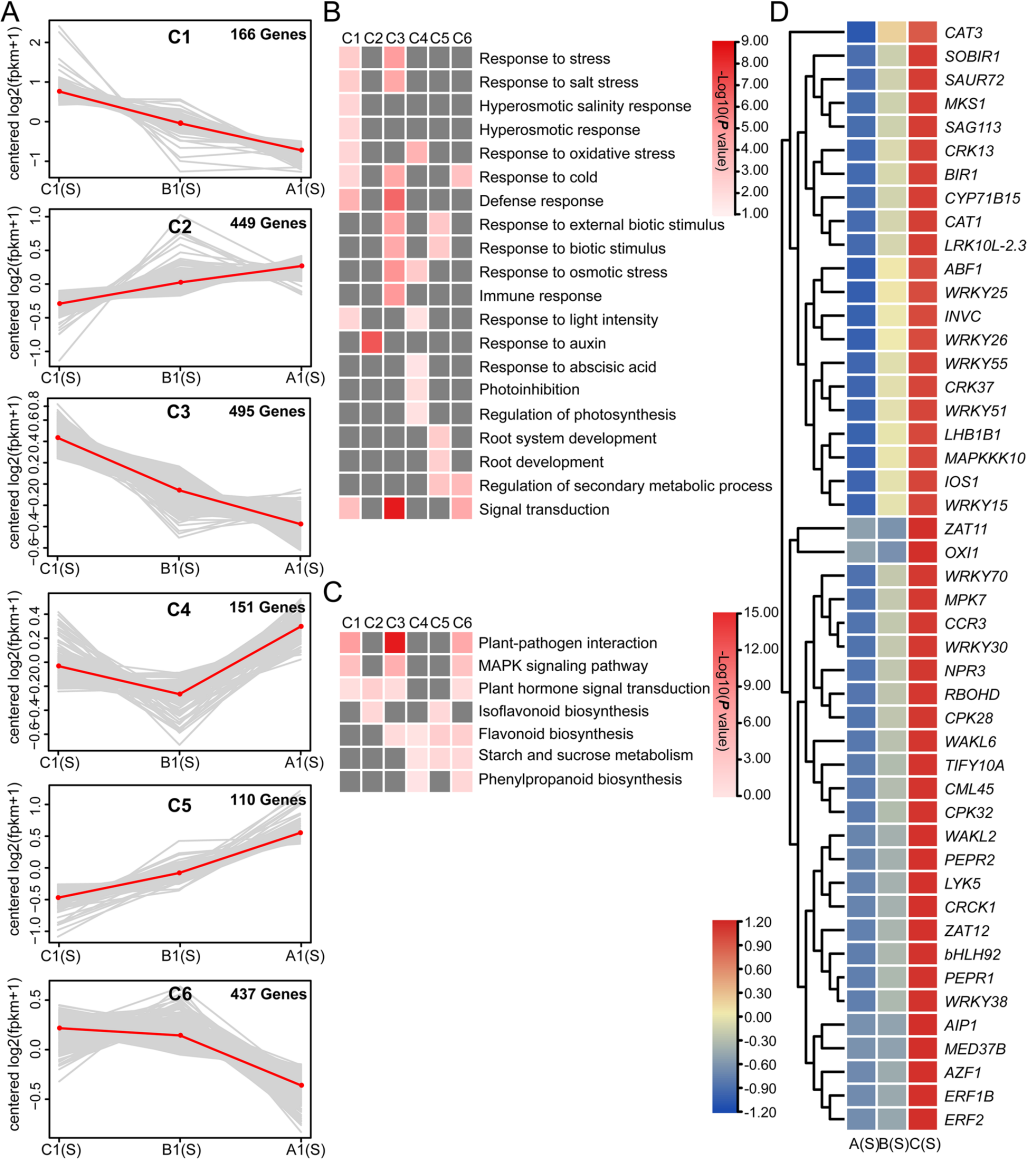
The dynamic profiles of DEGs in *Arabidopsis* in response to salt stress. **A** *K*-means clustering of the expression profile of DEGs. **B** Analysis of functional category enrichment among the six clusters. **C** Analysis of pathways enrichment among the six clusters. **D** The heatmap of DEGs related to abiotic stress response based on FPKM value (Z-score method). A: wild type (Col-0); B: *35S::bHLH*-overexpressing transgenic *Arabidopsis*; C: *P*_*bHLH*_*::bHLH*-overexpressing transgenic *Arabidopsis*; (S): salt treatment

To further elucidate the relationship between different *Arabidopsis* lines, DEGs under salt treatment were categorized into six groups by K-means analysis (Fig. 9A), implying changes in gene expression after overexpressing *35S::bHLH* or *P*_*bHLH*_*::bHLH*. According to the gene expression tendency, six groups were further divided into increasing (C1, C3, C6) and decreasing subgroups (C2, C4, C5). To determine the functional significance of the transcriptional changes of each group, GO classification and KEGG pathways were performed in analysis (Fig. 9B, C). DEGs in group 1 and 3 were enriched in stress responses (such as salt, cold, osmotic and external stimuli), immune response, defense response, and other biotic stimuli, which were mediated by the MAPK signaling pathway, plant hormone signal transduction pathway and plant-pathogen interaction; while group 4 and 5 were mostly involved in compound biosynthesis and metabolism (such as anthocyanin, flavonoid, phenylpropanoid, and isoflavonoid). The expressions of DEGs within group 1 were further analyzed, in which most of the abiotic stress-related genes were transcription factors and protein kinases (Fig. 9D). These results suggest that the function of upregulated DEGs, especially TFs, may be enhanced in salt response.

#### Gene co-expression analysis after salt stress by WGCNA (weighted gene co-expression network analysis)

**Fig. 10:**
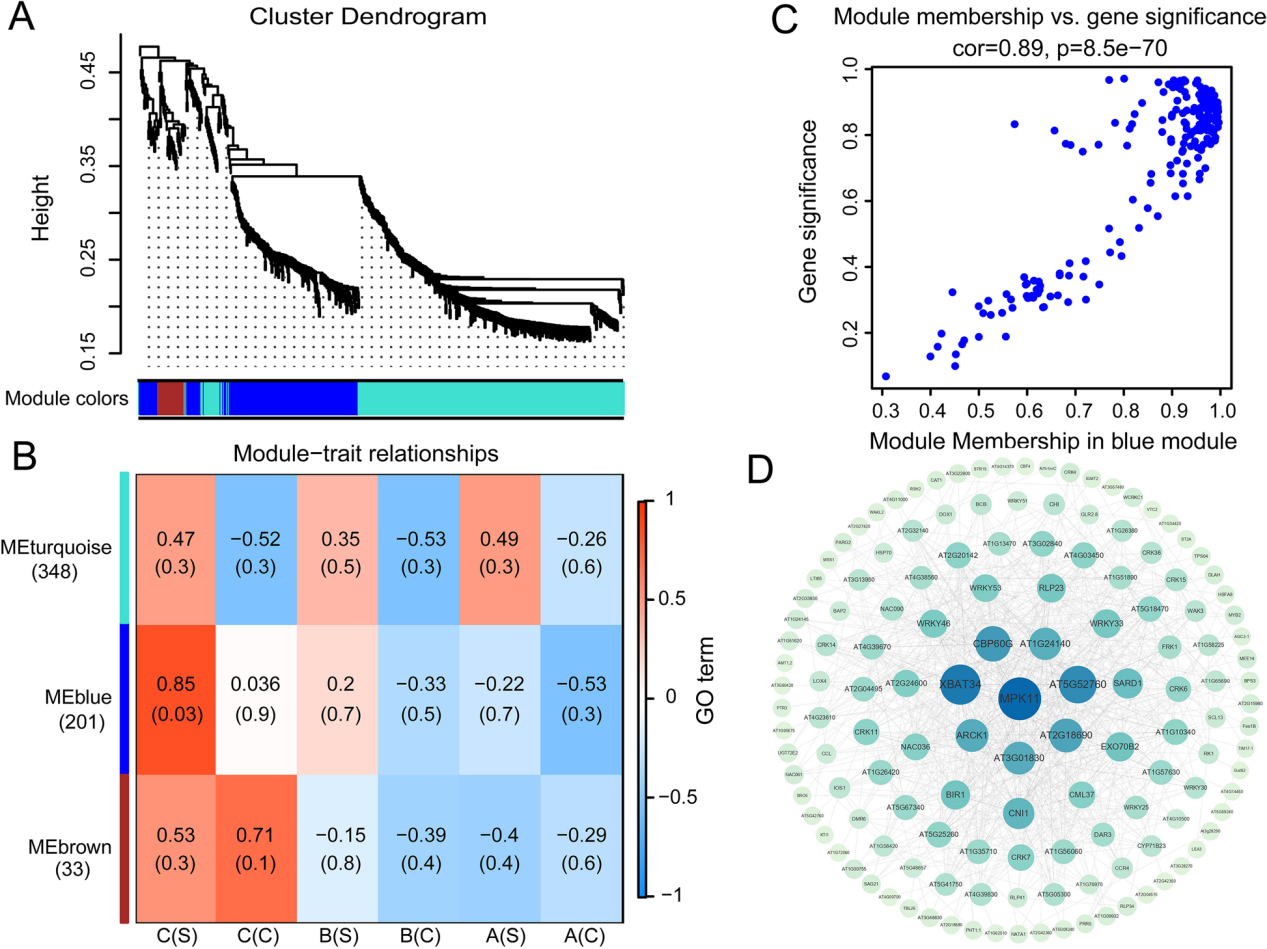
WGCNA of the transcripts in WT, *35S::bHLH* and *P*_*bHLH*_*::bHLH Arabidopsis* lines under salt treatment. **A** Gene dendrograms of the whole-transcriptome profiles were constructed using average linkage hierarchical clustering, each line represents one gene. The module color underneath the cluster tree shows the results of module assignment by the dynamic tree cut. **B** Correlations between modules eigengenes and different lines. The color of each module is the same as that in (**A**). The gene number of each module is shown in the bottom of the module name. The correlation coefficient and *p*-value are shown in each cell. **C** Scatterplots of gene significance versus module membership for the blue module. **D** Co-expression network of members in blue module. The largest inner circle represents the hub gene, and the relationships of all the genes are connected by lines. A: wild type (Col-0); B: *35S::bHLH*-overexpressing transgenic *Arabidopsis*; C: *P*_*bHLH*_*::bHLH*-overexpressing transgenic *Arabidopsis*; (C): normal condition; (S): salt treatment

To better understand the regulatory networks of *Arabidopsis* overexpressing *35S::bHLH* and *P*_*bHLH*_*::bHLH* in response to salt stress, 6 samples with three replicates and their expression data sets were used in WGCNA. A total of 3 co-expression modules were identified (threshold of similarity > 0.25, threshold of gene expression > 1), each module was marked in different color (Fig. 10A). The correlations between modules and samples were analyzed (Fig. 10B), the blue module positively correlated with the *P*_*bHLH*_*::bHLH* transgenic lines under salt treatment (*r* = 0.87, *p* = 0.03), implying a role in salt stress response. Notably, GO annotation of DEGs in blue module revealed that the top 20 of most enriched GO terms were involved in responses to salt stress, oxidative stress, wounding, defense, leaf senescence and other more (Fig. S5D in the Additional file 5). Scatterplots of gene significance (GS) and module membership (MM) in the blue module showed that both were highly correlated, indicating that the candidates were most significantly associated with the stress response of *P*_*bHLH*_*::bHLH* transgenic *Arabidopsis* (Fig. 10C). Furthermore, a hub gene MPK11 in this module was focused, which was directly related to plant-pathogen interaction and MAPK signaling pathway (Fig. 10D).

#### Hormone signal transduction and MAPK signaling under salt stress

**Fig. 11:**
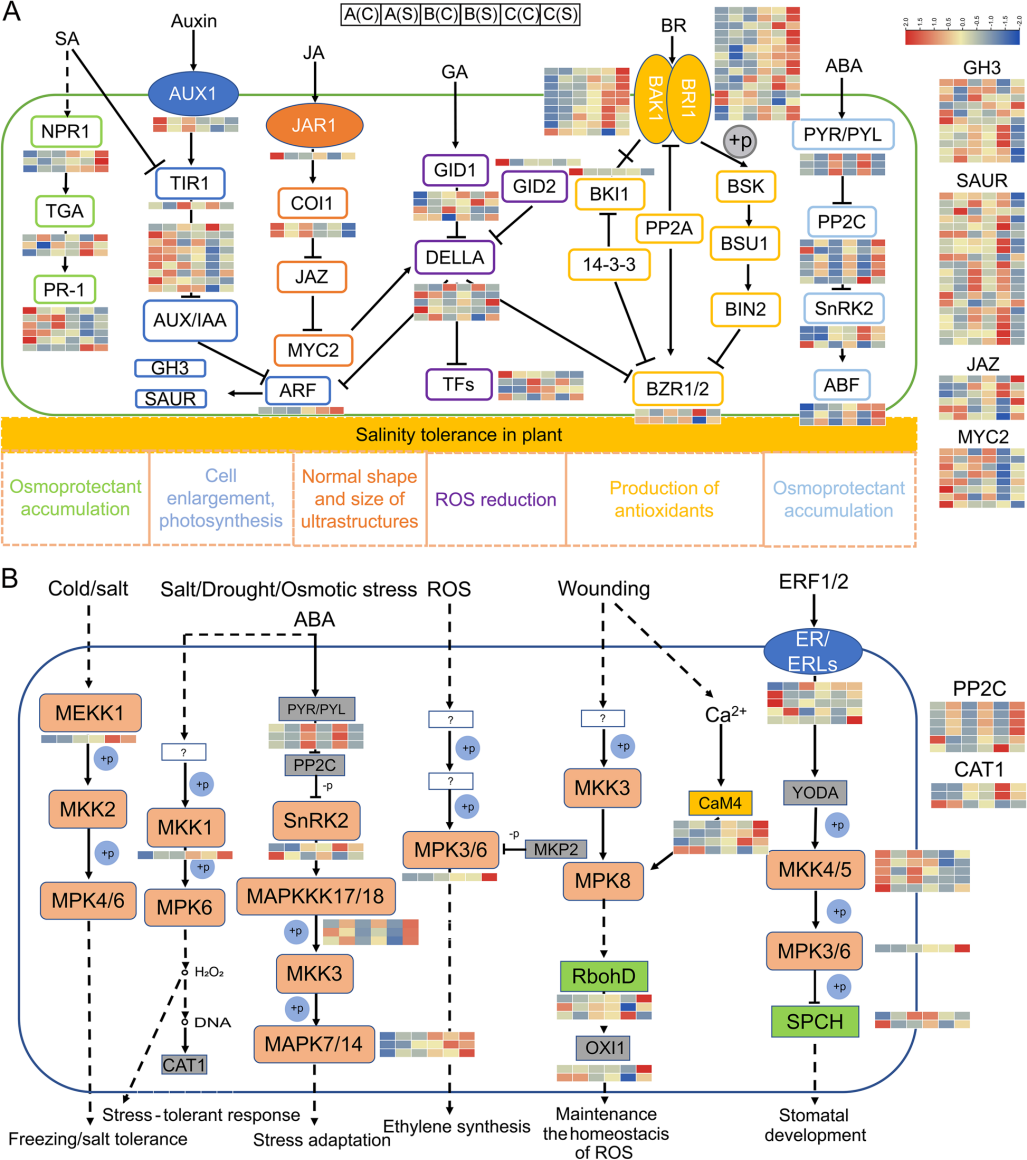
Transcriptional changes of DEGs involved in plant hormone signal transduction (**A**) and MAPK signaling pathway (**B**) to regulate plant in salt tolerance [25]. Blue circle with a ‘P’ inside indicates phosphorylation. A: wild type (Col-0); B: *35S::bHLH*-overexpressing transgenic *Arabidopsis*; C: *P*_*bHLH*_*::bHLH*-overexpressing transgenic *Arabidopsis*; (C): normal condition; (S): salt treatment

Our analyses showed that DEGs were significantly enriched in plant hormone signal transduction (ko04075) and MAPK signaling pathway (ko04016), which means that the main plant hormone-related genes must be activated. The results confirmed that three ABA receptor genes (PYR/PYL) were downregulated when subjected to salt stress, while the other components of the ABA signaling pathway (PP2Cs, SnRKs, and ABF TFs) were mostly upregulated in transgenic lines. *BAK1/BRI1* gene that encodes BR receptor was mostly upregulated in transgenic lines or under salt treatment. In SA signaling pathway, a total of 12 DEGs were identified, of which, 3 *NPR1* (nonexpressor of pathogenesis-related) genes were upregulated in transgenic lines under salt treatment, while multiple *PR-1* genes were downregulated in transgenic lines, especially in *P*_*bHLH*_*::bHLH* line. AUX/IAA, GH3 (Gretchen Hagen 3), and SAUR (small auxin-up RNA) are three different types of early auxin-responsive gene families. A number of DEGs related to auxin signaling were identified, and most of the auxin-responsive genes were downregulated in transgenic plants (Fig. 11A). In addition, GA receptor gene *GID1* (gibberellin insensitive dwarf1) was upregulated in transgenic lines under salt stress, meanwhile, a lot of DEGs participated in the GA signaling pathway. In the jasmonic acid (JA) signal transduction pathway, *JAR1* gene (jasmonic acid-amido synthetase) was downregulated in transgenic lines while upregulated under salt treatment, *COI1* gene was downregulated in transgenic lines or under salt treatment, *JAZ* and *MYC* genes were upregulated in salt-treated transgenic lines. Protein kinases (PKs) in the MAPK signaling cascade, such as MEKK1, MKK1, MAPKKK17/18, MPK3/6, and MAPK7/14, showed significantly increased expression in transgenic lines under salt treatment, and a better performance was observed in *P*_*bHLH*_*::bHLH* transgenic *Arabidopsis*; while MKK4/5 decreased when suffered from salt stress (Fig. 11B). The above data and analyses indicate that multiple phytohormones and the MAPK signaling pathway may have important contribution to the regulatory mechanisms associated with salt stress tolerance in transgenic *Arabidopsis*.

### Validation of the key genes in transgenic *Arabidopsis* by qRT-PCR

**Fig. 12:**
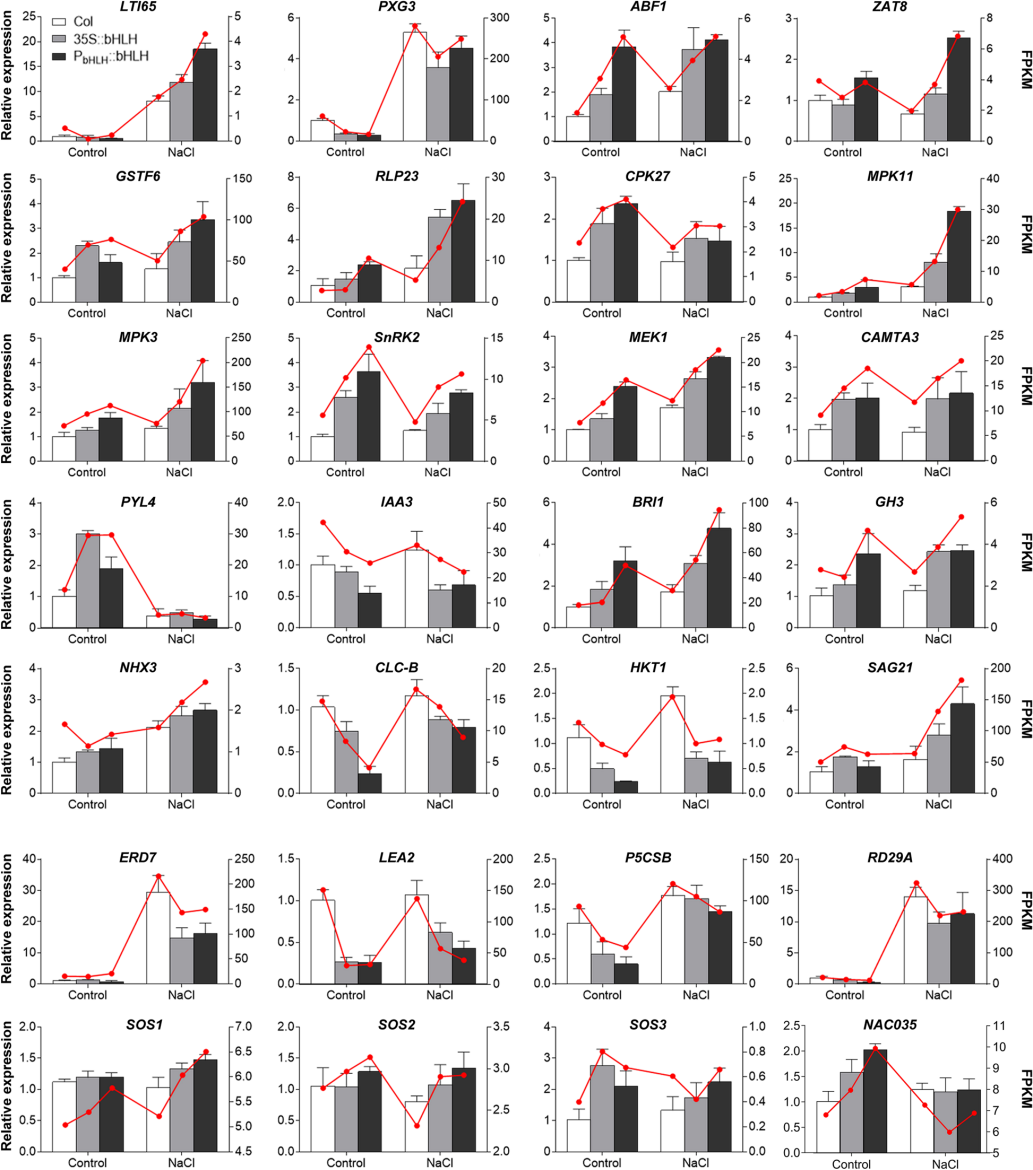
Changes in relative expression level of DEGs analyzed by qRT-PCR and RNA-seq. The left vertical axis indicates the relative expression level determined by qRT-PCR (bar chart), the right vertical axis indicates the FPKM value determined by RNA-seq (line chart). *LTI65*, low temperature induced 65 kD protein; *PXG3*, peroxygenase 3; *ABF1*, ABRE binding factor; *ZAT8*, zinc finger of *Arabidopsis thaliana* 8; *GSTF6*, glutathione S-transferase 6; *RLP23*, receptor like protein 23; *CPK27*, calcium-dependent protein kinase 27; *MPK3/11*, mitogen-activated protein kinase 3/11; *SnRK2*, serine/threonine-protein kinase 2; *MEK1*, MAP kinase/ ERK kinase 1; *CAMTA3*, calmodulin-binding transcription activator 3; *PYL4*, PYR1-like 4; *IAA3*, auxin-responsive protein IAA3; *BRI1*, brassinosteroid insensitive 1; *GH3*, auxin-responsive GH3 family protein 3; *NHX3*, Na^+^/H^+^ antiporter; *CLC-B*, chloride channel protein CLC-B; *HKT1*, high-affinity K^+^ transporter 1; *SAG21*, senescence-associated gene 21; *ERD7*, early responsive to dehydration 7; *LEA2*, late embryogenesis abundant protein; *P5CSB*, delta 1-pyrroline-5-carboxylate synthase 2; *RD29A*, desiccation-responsive protein 29A; *SOS1, 2, 3*, salt overly sensitive 1, 2, 3; *NAC035*, NAC domain containing protein 35

The expression profiles of 28 genes randomly selected from the identified stress-related DEGs were analyzed by quantitative RT-PCR to compare with their transcriptomic data, including TFs, PKs, ion transporters, hormone- or stress-related genes (Table S5 in the Additional file 12). Among them, TFs and PKs in transgenic lines or under salt treatment were all upregulated. The expression levels of hormone-related genes significantly fluctuated, *BRI1* and *GH3* were upregulated in transgenic lines or under salt treatment, while *IAA3* was downregulated. For the ion transporters, the expression levels of *CLC-B* (chloride channel protein) and *HKT1* (high-affinity K^+^ transporter) were lower in the transgenic plants than in the WT, while increased under salt stress. *NHX3* transcripts were significantly accumulated after salt treatment. All test stress-responsive genes were upregulated under salt treatment. The expression patterns of these DEGs were highly consistent with the FPKM values of RNA-seq data (Fig. 12), indicating that the analyses of RNA-seq-based transcriptional profiles are reliable and all these key genes are involved in salt stress tolerance.

## Discussion

### *CgbHLH001* promoter and functional region can respond to abiotic stress

As the key controller of gene expression, the promoter consists of diverse *cis*-acting elements, motifs, and other regulatory sequences [26]. Many functional *cis*-elements distributed in the 5’ regulatory region of genes may involve in the transcriptional regulation to control different biological processes and respond to different stimuli [27–29]. Therefore, characterization of the functional elements and promoter activity is necessary for understanding of gene expression regulation and application in genetic engineering. In the present study, a 1512 bp promoter sequence of *CgbHLH001* gene was identified, which displayed a strong driving activity and was inducible under abiotic stress; the 5’ UTR sequence was necessary to drive the promoter activity.

*Cis*-regulatory elements can largely control the precise sensitivity and specificity of transcriptional responses [30]. ABRE is known as ABA-responsive element, which is also a *cis*-acting element on dehydration, high salinity and low temperature responsiveness [4]. ABRE motif in *GmRD26* promoter in *Glycine max* applies a strong induction under drought condition to trigger its downstream gene expression in response to stress [31]. This element in *FeDREB1* (dehydration-responsive element binding protein) promoter from *Fagopyrum esculentum* can activate cold- and drought-responsive gene expression [32]. In the present study, a number of *cis*-acting elements were predicted in *CgbHLH001* promoter, and many associated with phytohormone, light and abiotic stress responsiveness, such as ABRE, G-box, W-box, MYB recognition site, and MYB binding site, which might activate *CgbHLH001* when subjected to NaCl, PEG, ABA, MeJA and light treatment in *C. glaucum*. In accordance with the element distribution in *CgbHLH001* promoter, the full length (FL) promoter (1512 bp) presented quite high activity, which was gradually reduced with 5’ stepwise deletion increasing. Our results suggest that *cis*-elements distributed in *CgbHLH001* promoter apply apparent effects on promoter activity.

It has been reported that 5’ UTR plays important roles in regulation of gene expression at transcriptional or/and post-transcriptional level(s) [33]. The changes of 5’ UTR structure in *Arabidopsis* may result in an over 200-fold variation in mRNA stability and translational efficiency [34–36]. 5’ UTR of *RBCL* (rubisco large subunit) gene in tobacco can compensate for the low-rate transcription by enhancing the mRNA stability in the dark [37]. And also, some 5’ UTRs from higher plants can act as translational enhancer [38–40]. The 5’ UTR of *AtCOR47* (cold-regulated 47) gene acted as an effective translational enhancer to ensure the stable and high expression of gene under different conditions [6]. In the present study, the absence of 5’ UTR from the *P*_*bHLH*_* FL* promoter resulted in approximate eightfold reduction of gene expression, suggesting that the 5’ UTR (364 bp) applies a positive effect on the promoter activity. Various regulatory elements, the secondary structure of mRNA and *trans*-factor accessibility in 5’ UTR all impact on the expression of downstream open reading frame [41]. The 5’UTR RNA sequence of *PtDrl02* gene forms a stable secondary structure with -45.1 kcal mol^−1^ of the folding free energy (∆G), resulting in decreases of *GUS* mRNA expression and GUS activity [42]. In the present study, we found that 5’ UTR of *CgbHLH001* presented much complicated secondary RNA structure with a ∆G value of -81.13 kcal mol^−1^, which resulted in significant increases at the transcription level and translation efficiency of *GUS*. Further analysis revealed a Py-rich stretch sequence in this region, which is known to promote gene transcription at high level [43]. Taken together, our results suggest that the 5’ UTR sequence combined with its structure may confer a positive regulatory effect on *CgbHLH001* gene expression at transcriptional and translational levels.

### Overexpressing *P*_*bHLH*_*::bHLH* enhances tolerance to abiotic stress

An increasing number of bHLH TFs have been reported to play an essential role in response to abiotic stress [44–46]. Ectopic expression of *Pyrus ussuriensis PubHLH1* results in a higher survival rate of transgenic tobacco than WT under chilling treatment [47]. We previously identified a *bHLH* gene (*CgbHLH001*) from a halophyte *C. glaucum* [23], which conferred drought tolerance to transgenic tobacco and maize [48], moreover, enhanced photosynthetic capacity was also achieved in *CgbHLH001*-overexpressing maize. In the present study, the transgenic *Arabidopsis* overexpressing *P*_*bHLH*_*::bHLH* presented a better performance than the WT in relevant gene expression at transcriptional and translational levels when suffered from salt, drought or lower temperature stress. Our results suggest that *CgbHLH001* promoter is inducible and can drive *CgbHLH001* overexpression ectopically, which in turn enhances stress tolerance in transgenic *Arabidopsis*. Although *CgbHLH001* gene was expressed at a higher transcriptional level in transgenic *Arabidopsis*, while much lower translational level was present, regardless the *CaMV35S* or *CgbHLH001* promoter was used. Accumulating evidence suggests a mismatch between transcriptional and translational levels, and the obvious difference between mRNA level and protein expression may be attributed to post-transcriptional regulation and/or post-translation modifications [49]. Besides, the apparent difference of the CgbHLH001 protein level was also found between the ectopic expression in *Arabidopsis* (much lower level) and ontologic expression in *C. glaucum* (relatively higher level; Fig. 4B) under the same condition. It may be the cytosol environment in *Arabidopsis* (glycophyte) unfavorable for CgbHLH001 protein (originated from halophyte) accumulation, together with the reason that the protein expression efficiency is determined by multiple factors, including translation rates [50], translation modulation [51], protein’s half-life [52], protein synthesis delay and protein transport [53].

### Signaling networks regulated by CgbHLH001 TF responds to abiotic stress

In plant stress responses, receptors or sensors recognize stress signals, which are in turn transmitted via secondary messengers (Ca^2+^, hormone, and ROS). Various physiological responses are further triggered by signal transduction, in which PKs (CDPKs, MAPKs) control downstream TFs and then regulate the expression of target genes that may ultimately influence the stress tolerance of plants [54]. Based on our analysis, the hormone signal transduction and MAPK signaling pathway were involved in regulating downstream gene network of TF-mediated stress responses, such as CgbHLH001 TF. In the process, multiple PK, TFs and stress-related genes were significantly induced, which may participate in multiple signaling pathways and respond to external stress.

Plant hormone signal transduction is an essential component of plant stress-response signaling pathways, as the most important endogenous substances, phytohormones play critical roles in stress response and growth promotion [55]. The transcriptome analysis of *Podocarpus macrophyllus* indicate that the transcription of genes involved in biosynthesis and phytohormone signaling pathways have been altered significantly, and genes related to auxin transport and responsiveness are downregulated in response to salt stress [56]. In *Chenopodium quinoa*, salt stress results in a decrease in signaling components of growth-related phytohormones (auxin, BR) and an increase of components in ABA signaling pathway (PP2Cs), while some other important components of ABA pathway (PYLs, SnRK) are downregulated under long-term salt stress [57]. As an important stress-responsive hormone, ABA plays indispensable roles in regulation of the balance of osmosis, ions, and ROS under salt stress [58]. In the present study, the ABA receptor genes *PYR/PYL* were significantly upregulated in two types of transgenic *Arabidopsis* compared to WT. Protein phosphatase 2C (PP2C) is another ABA signaling pathway related gene known to participate in the stress response [59], our results showed a significant increase of *PP2C* in transgenic *Arabidopsis* when suffered from salt stress, which finally activated the expression of its substrate *ABFs*. SA also plays an important role in plant stress tolerance by participating in the accumulation of osmoprotectants and induction of antioxidant enzymes under salt stress [60]. Increase in SA level results in the induction of *PR* (PATHOGENESIS RELATED) genes, and the *NPR1* (NON-EXPRESSOR OF PR GENE 1) functions as the key regulatory elements in SA-dependent activation of *PR* genes [61]. Our results revealed that *NPR1* was induced in two types of transgenic *Arabidopsis* under salt stress, while the *PR-1* was mostly downregulated (Fig. 11). Further exploration of the phytohormone signal transduction and the crosstalk between different gene signals at the physiological levels may partly explain the mechanisms in stress tolerance.

Genes encoding PKs, such as receptor like kinase-Pelle (RLK-Pelle), CDPK, and MAPK, were largely induced among multiple comparisons (Fig. S7 in the Additional file 7). Our DEGs data suggest that MAPK signaling pathway was highly enriched with upregulated genes in transgenic *Arabidopsis* under salt treatment (Figs. 6B and 11B). RLK-Pelle is the largest gene family in *Arabidopsis* and rice, which are responsible for multiple abiotic and biotic stresses [62]. In the present study, the RLK-Pelle kinases accounted for much high proportion, especially in [A(C) *vs* C(C)] and [A(C) *vs* B(C)], indicating that PKs can be one of the important factors associated with salt tolerance after overexpressing *CgbHLH001* gene (Fig. S7 in the Additional file 7).

TFs are activated after perceiving stress signals, the downstream related genes are subsequently regulated to respond to external stress [63, 64]. Numerous upregulated TFs have exclusively been detected in salt-tolerant genotype of barley, including AP2/ERF, bZIP, MYB-related, WRKY, Trihelix, and bHLH, which suggests the crucial roles of these TFs in regulating the downstream genes responsible for salt stress tolerance [65]. Transcriptome analysis between drought-tolerant (Otis) and drought-sensitive (Baronesse) barley genotypes showed that DEGs specifically induced or greatly upregulated under drought stress in Otis but not in Baronesse were important for drought tolerance [66]. In the present study, transcriptome analysis revealed dramatic changes in amount of TFs and AP2/ERF-ERF, and the next ones were NAC, bHLH, WRKY, MYB, C2H2, bZIP and other more, which may alleviate salt-induced damage (Fig. S7 in the Additional file 7). AP2/ERF-ERF genes are plant-specific TFs involved in growth/development and stress responses [67–70]. Here, abundant DEGs of AP2/ERF-ERF TF were identified in transgenic *Arabidopsis* compared to WT, less differences were found between two types of transgenic *Arabidopsis* [B(C) *vs* C(C)]. The proportion of each TF family in different comparisons varied, indicating that the *CgbHLH001* (promoter and gene) in transgenic *Arabidopsis* may play important role in activating related TFs and functional genes in response to salt stress, and the participation of various PKs and TFs demonstrated that the kinase-mediated signal cascades were involved in salt stress response, which is consistent with our GO and KEGG enrichment analyses (Figs. 6 and 9). Furthermore, the downstream functional genes in signal transduction pathway were significantly upregulated under salt treatment (Fig. 12). However, the relationship between CgbHLH001 and its downstream functional genes still needs to be elucidated.

## Conclusion

Based on the previous characterization of the function of CgbHLH001 TF in halophyte *C. glaucum* [23], we further investigated the promoter activity and biological function of *CgbHLH001* gene associated with abiotic stress. In the present study, *CgbHLH001* promoter exhibited substantially higher activity and positive response to various abiotic stress, with the 5’ UTR acting as an enhancer of gene expression. Furthermore, *CgbHLH001* promoter induced ectopic overexpression of its own gene at transcriptional and translational levels, which results in stress tolerance to transgenic *Arabidopsis*. The transcriptome data suggest that the stress response of overexpressing *CgbHLH001* was involved in multiple biological processes. Here, a large number of DEGs were identified in transgenic *Arabidopsis* under salt stress, some of which were involved in plant hormone signal transduction and MAPK signaling pathway. Collectively, our findings provide a new insight into the regulatory function of *CgbHLH001* promoter and the TF in response to abiotic stress, and suggest a number of candidate genes that can potentially be used in developing stress tolerance crops in the future.

## Methods

### Plant materials, growth conditions and treatments

Mature seeds of *C. glaucum* were collected from natural plants growing at the edge of the Gurbantunggut desert at Wujiaqu 103 regiment (44°37'N, 87°26'E; 423 mH) in October 2014, in the Xinjiang Uygur Autonomous Region, China. After collection, the plant was identified by Yongman Lu (a plant taxonomist in Xinjiang University), and voucher specimens (No. CG201410) was deposited at the herbarium (College of Life Science and Technology, Xinjiang University). Seeds were air-dried indoor and cleaned and then stored at 4ºC in sealed brown-paper bags. Wild type (WT, Columbia-0) and transgenic lines of *Arabidopsis thaliana*, and *Chenopodium glaucum* were cultivated in pots containing a mixed soil (peat soil: vermiculite = 3:1, v/v) under the greenhouse condition of Xinjiang University, China: at 22ºC (*A. thaliana*) or 25ºC (*C. glaucum*), 20–30% relative humidity (RH), 16 h light/8 h dark photoperiod, 100 μmol m^−2^ s^−1^ light intensity. Seedlings (plants) were well-watered and applied with half-strength Hoagland solution [71] at an interval of two weeks.

For phenotypic analysis, 3-week-old seedlings of WT and T3 transgenic *Arabidopsis* lines harboring *35S::bHLH* or *P*_*bHLH*_*::bHLH* were subjected to abiotic stress with 200 mM NaCl treatment for 15 d, natural drought treatment for 20 d or 4ºC treatment for 14 d. Four replicates of each line with 5 plants of each replicate were applied.

For immunoblotting analysis, 4-week-old seedlings of transgenic *Arabidopsis* or *C. glaucum* were treated under normal condition (applied with Hoagland solution) or abiotic stress [200 mM NaCl, 300 mM mannitol, 10% PEG treatments (prepared with Hoagland solution), and 4ºC treatment] for 24 h, and sampled for protein extraction.

For transient expression analysis, 2-week-old seedlings of *C. glaucum* were used in *Agrobacterium* infiltration.

For qRT-PCR analysis of *CgbHLH001* in *C. glaucum*, (1) NaCl or PEG 6000 stress: mature seeds were sown on two layers of filter paper in a 9 cm Petri dish, which were saturated with 5 mL of different concentrations of solution—NaCl (0, 50, 100, 300 mM), or PEG 6000 (0, 5, 10, 15, 20%), and sealed with cling film in an incubator (25ºC, 30–40% RH, 16 h light/8 h dark, 100 μmol·m^−2^·s^−1^ light intensity), 2-week-old seedlings were sampled. (2) Phytohormone treatment: 2-week-old seedlings grown on MS medium (Coolaber, Beijing, China) were carefully transferred into MS solution in addition with ABA (0, 1, 2, 5, 10 μM), GA_3_ (1, 5, 10, 20 mg·L^−1^), or MeJA (1, 5, 10, 20 μM), and shaken at 30–50 rpm, 25ºC for 2 h (ABA), or 5 h (GA_3_ and MeJA), and then sampled. (3) Different light quality treatment: mature seeds were sown on two layers of filter paper (saturated with 5 mL ddH_2_O) in a 9 cm Petri dish, which was then sealed in a colored transparent box [blue (450–490 nm), green (491–570 nm), yellow (570–750 nm), red (621–750 nm)], the foil wrapped Petri dish was used as darkness treatment. All Petri dishes were placed under normal light (380–750 nm wavelength) in a plant incubator (25ºC, 30–40% RH, 24 h constant light, 100 μmol·m^−2^·s^−1^ light intensity). Samples were collected at 0, 1, 3, 5, 7 days after seeds were sown. Three biological replicates for each treatment were applied to all treatments. All samples were frozen immediately at –80ºC upon harvesting for further use.

### Cloning and sequence analysis of *CgbHLH001* promoter

Genomic DNA of *C. glaucum* was isolated using DNAsecure Plant Kit (Cat. DP320; Tiangen Biotech., Beijing, China). *CgbHLH001* promoter fragment was achieved by using Genome Walking Kit (Cat. 6108; TaKaRa, Dalian, China). Gene specific primers were shown in Table S6 in the Additional file 13. After sequencing, the *cis*-acting elements were predicted by PlantCARE (http://sphinx.rug.ac.be:8080/PlantCARE/). The transcription start site (TSS) of the promoter was predicted by TSSP-Prediction of PLANT Promoters (http://linux1.softberry.com/berry.phtml?topic=tssp&group=programs&subgroup=promoter). Secondary structures of 5’ UTR were predicted by Mfold RNA/DNA folding software (http://www.bioinfo.rpi.edu/applications/mfold/).

### Total RNA extraction and quantitative RT-PCR analysis

Total RNA was isolated from seedlings or plant tissues using Plant RNA Extraction Kit (Omega, USA) according to the manufacturer’s instructions. The reverse transcription reaction was performed with 1.0 μg of total RNA in a volume of 20 μL using the reverse transcriptase M-MLV (TaKaRa, Dalian, China). qRT-PCR was performed using the PerfectStart Green qPCR SuperMix (Cat. AQ601; TransGen, Beijing, China) in a LightCycler 96 Real-Time System (Roche, United States). The reactions were conducted in a 20 μL volume of mixture (containing 10 μL of 2 × SuperMix, 0.4 μL of 10 mM of each primer, 8.2 μL of ddH_2_O, and 1 μL of cDNA) at conditions: 94ºC 30 s; 40 cycles of 94ºC 5 s, 60ºC 30 s. *CgGAPDH* (for *C. glaucum*) or *Atactin* (for *Arabidopsis*) was used as an internal reference to normalize the expression level. Primers used for qRT-PCR were shown in Table S6 in the Additional file 13. Three biological replicates with two technical replicates of each were applied. The relative expression level of each gene was calculated by 2^−ΔΔCT^ method [72].

### Transient expression assay mediated by *Agrobacterium*

Single colonies of *A. tumefaciens* GV3101 harboring pCAMBIA1304-*35S*::*GUS* and pCAMBIA1300-*P*_*bHLHs*_::*GUS* [*P*_*bHLHs*_ represent: full length and truncated segments (960 bp, 521 bp, and 364 bp upstream of the ATG codon) of the promoter; 1148 bp upstream of the TSS] were cultured in YEB liquid medium (50 mg·L^−1^ kanamycin, 40 mg·L^−1^ gentamicin and 50 mg·L^−1^ rifampicin) at 28ºC with shaking for overnight. Cultures (200 μL) were transferred to 20 mL fresh YEB medium and incubated at 28ºC with shaking till OD_600_ = 0.8, then harvested by centrifugation at 8000 rpm for 10 min, after being resuspended, the *Agrobacterium* slurry was adjusted to OD_600_ of 0.8 with 1/2 MS solution [containing 120 μM acetosyringone, 2.5% (w/v) sucrose, and 0.01% (w/v) Tween 20, pH 5.8] and employed for the transient transformation, pCAMBIA1300-GUS was used as control. Two-week-old seedlings of *C. glaucum* were pre-treated for hyperosmosis in 1/2 MS solution [containing 25% (w/v) sucrose, pH 5.8] for 2 h, and then submerged in transformation solution and shaken at 100 rpm at 28ºC for 3 h. Treated seedlings were washed with ddH_2_O for five times, and then vertically inserted in 1/2 MS solid medium [containing 120 μM acetosyringone, 2.5% (w/v) sucrose, pH 5.8] for co-cultivation. Three days later, seedlings were sampled for GUS staining and qRT-PCR analysis.

### Genetic transformation and generation of transgenic lines of *A. thaliana*

T0 seeds of *A. thaliana* transformed by the floral dip method [73] were harvested and screened on solid MS medium containing 30 mg·L^−1^ hygromycin, T3 generation transgenic lines of pCAMBIA1304-*35S::GUS*, *P*_*bHLH*_::*GUS FL*, *P*_*bHLH*_::*GUS 1148* and *P*_*bHLH*_::*GUS 364* were generated and used for GUS analysis (including gene expression, histochemical staining and enzyme activity) under various treatments. Two-week-old seedlings were transferred to 1/2 MS medium containing 200 mM NaCl, 200 mM mannitol, 2 μM ABA, or 10 μM MeJA for 2 d, 2 d, 2 h, or 5 h, respectively. Seedlings grown on 1/2 MS only were used as control. Photographs were captured by stereomicroscope (Nikon SMZ25, Japan). T3 generation transgenic *A. thaliana* lines harboring *35S::bHLH* or *P*_*bHLH*_*::bHLH* were generated by similar way and used for phenotypic analysis, gene expression and protein extraction.

### Analysis of GUS histochemical staining and GUS enzyme activity

For GUS staining, 2-week-old seedlings were immersed in staining solution [containing 1 mM X-Gluc, 100 mM sodium phosphate, pH 7.0; 1 mM potassium ferricyanide (K_3_Fe(CN)_6_), 1 mM potassium ferrocyanide (K_4_Fe(CN)_6_), 10 mM EDTA, pH 8.0; 0.1% Triton X-100] (prepared just before use and stored in the dark). Samples were treated in the dark in a shaker at 100 rpm at 37ºC overnight. After washed in ddH_2_O for 2–3 times, seedlings were boiled in de-staining solution (containing glacial acetic acid: anhydrous ethanol = 3:1) for 10 min till became completely transparent, then photographed. Fluorometric GUS activity was detected using 4-methyl-umbelliferyl-β-D-glucuronide (4-MUG) as the substrate. Samples (0.1 g) were harvested and homogenized in extraction buffer (Cat. SL7161, Coolaber, Beijing), after being centrifuged at 12,000 rpm for 10 min, aliquots of supernatant were incubated for 10, 20 min at 37ºC in extraction buffer containing 1 mM 4-MUG. The reaction was terminated by addition of 0.2 M Na_2_CO_3_. Fluorescence was then measured on a fluorescent spectrophotometer (F97pro, Shanghai) with 4-methylumbelliferone (4-MU) as standard. Protein concentration was measured using Bradford Kit (Cat. SK1060, Coolaber, Beijing) with bovine serum albumin (BSA) as standard.

### Protein extraction and detection in *C. glaucum* and transgenic* Arabidopsis*

The total protein of *C. glaucum* or transgenic *Arabidopsis* (with *35S::bHLH* or *P*_*bHLH*_*::bHLH*) was extracted using Protein Extraction Kit (Cat PTE001, Coolaber, Beijing). Plant tissues were ground in liquid nitrogen and then transferred into extraction buffer, followed by centrifugation at 12,000 rpm, 4°C for 15 min. The supernatant was used as crude protein solution, the protein concentration was determined by BCA (bicinchoninic acid) Protein Assay Kit (BCAP-2-W, Comin, Jiangsu). The crude protein was mixed with 4 × loading buffer and boiled for 10 min, then used for SDS-PAGE after centrifugation at 12,000 rpm for 10 min. Protein samples (60 μg of each) were resolved by 12% polyacrylamide gel and then transferred to a polyvinylidene fluoride membrane for immunoblotting analysis. The monoclonal mouse antibody against CgbHLH001 protein was developed (Abmart, Shanghai) and used at 1:500 dilution. β-tubulin (1:5000 diluted; M30109F, Abmart, Shanghai) served as internal control. The secondary antibody [Goat Anti-Mouse IgG (H + L) conjugated horseradish peroxidase, TransGen, Beijing] diluted at 1:10,000 for detection. The target protein was visualized by enhanced chemiluminescence (EasySee Western Blot Kit, TransGen, Beijing), and images were acquired by luminescent image analyzer (Amersham Imager 600, GE, USA).

### RNA-Sequencing analysis

Three-week-old plants of WT and transgenic *Arabidopsis* overexpressing *35S::bHLH* or *P*_*bHLH*_*::bHLH* (treated with either normal water or 200 mM NaCl for 1 h) were used for RNA sequencing. High-quality RNA was used (RNA integrity number (RIN) ≥ 8.5, OD_260_/_280_ ≥ 1.9 and OD_260_/_230_ ≥ 1.5). Sequencing was performed on an Illumina HiSeq2000 Platform (BioMarker technologies Co., Ltd, Beijing, China). Three independent biological replicates were sequenced. For the raw data, fastp v0.21.0 was used to filter adapter, low-quality base and low-complexity reads [74]. Parameters were set as follows: -q (qualified_quality_phred) = 10; -u (unqualified_percent_limt) = 50; -g (trim_poly_g) = 10; -Y (complexity_threshold) = 10; -e (average_qual) = 20; -l (legth_required) = 100; -b (max_len1) = 150; -B (max_len2) = 150. After filtering the raw data, all clean reads were aligned to *Arabidopsis* reference genome (ftp://ftp.ensemblgenomes.org/pub/plants/release-45/fasta/arabidopsis_thaliana) using Hisat2 v2.0.4 software (JHU, Baltimore, USA) (http://ccb.jhu.edu/software/hisat2/index.shtml). The mapped reads were assembled and merged using StringTie v1.3.4d software (JHU, Baltimore, MD, USA) (https://ccb.jhu.edu/software/stringtie/index.shtml). DEGs were identified using DEseq2 software (false discovery rate < 0.05 and FC ≥ 1.5). Gene Ontology (GO) enrichment analysis of DEGs was implemented via GOseq R packages v3.10.1 based on Wallenius non-central hyper-geometric distribution. Gene function was annotated using six primary databases: National Center for Biotechnology Information (NCBI), non-redundant protein sequences (NR), Kyoto Encyclopedia of Genes and Genomes (KEGG), manually annotated and commented protein sequence (Swiss-Prot), protein family (Pfam) and Gene Ontology (GO). Protein–protein interactions were analyzed using STRING software. Cytoscape software was used to visualize co-expression networks. Weighted gene co-expression network analysis (WGCNA) was used to describe correlation patterns among genes across multiple plants on the platform BMKCloud (www.biocloud.net). The heatmaps were drawn using the TBtools [75]. qRT-PCR of 28 DEGs that were critical to salt stress response was performed to validate the RNA-seq data. *AtActin* as the reference gene, all the primers used are listed in Table S6 in the Additional file 13.

### Statistical analysis

All data were expressed as means ± SD. One-way ANOVA was used to test the significance of main effects, and Tukey’s test was performed for multiple comparisons to determine significant differences between samples at 0.05, 0.01 or 0.001 significance level.

## Supplementary Information

**Additional file 1: Fig. S1.** Phenotype performance and gene expression of transgenic Arabidopsis lines overexpressing *35S::bHLH* and *P*_*bHLH*_*::bHLH* in response to drought stress. **A** Transcriptional expression of *CgbHLH001* gene. **B** Translational expression of *CgbHLH001* gene. **C**-**D** Phenotypic observation and survival percentage of transgenic Arabidopsis. OE35S1, 2: *35S::bHLH*-overexpressing transgenic line 1, 2; *OEPb1*, 2: *P*_*bHLH*_*::bHLH*-overexpressing transgenic line 1, 2. Different lowercase letters in a indicate significant difference existing between different transgenic lines.

**Additional file 2: Fig. S2.** Phenotype performance and gene expression of transgenic Arabidopsis lines overexpressing *35S::bHLH* and *P*_*bHLH*_*::bHLH* in response to 4°C treatment. **A** Transcriptional expression of *CgbHLH001* gene. **B** Translational expression of *CgbHLH001* gene. **C**-**D** Phenotypic observation and survival percentage of transgenic Arabidopsis. *OE35S1*, 2: *35S::bHLH*-overexpressing transgenic line 1, 2; *OEPb1*, 2: *P*_*bHLH*_*::bHLH*-overexpressing transgenic line 1, 2. Different lowercase letters in **A**, **D** indicate significant difference existing between different transgenic lines.

**Additional file 3: Fig. S3.** The heatmap of correlation analysis (**A**) and principal component analysis (**B**) among replicates in the same group and between different groups. A: wild type (Col-0); B: *35S::bHLH*-overexpressing transgenic Arabidopsis; C: *P*_*bHLH*_*::bHLH*-overexpressing transgenic Arabidopsis; (C): normal condition; (S): salt treatment.

**Additional file 4: Fig. S4.** The most enriched GO terms in different comparisons. **A**-**B** 542 upregulated and 494 downregulated DEGs in A(C) *vs* C(C); **C**-**D** 88 upregulated and 207 downregulated DEGs in A(C) *vs* B(C); **E**-**F** 182 upregulated and 415 downregulated DEGs in the overlap between A(C) *vs* C(C) and A(C) *vs* B(C). A: wild type (Col-0); B: *35S::bHLH*-overexpressing transgenic Arabidopsis; C: *P*_*bHLH*_*::bHLH*-overexpressing transgenic *Arabidopsis*; (C): normal condition.

**Additional file 5: Fig. S5.** The most enriched GO terms in various comparisons. **A** The most enriched GO terms in the top 50 DEGs with the highest fold change in comparison B(S) *vs* C(S). **B** The most enriched GO terms in the top 50 DEGs with the highest fold change in comparison A(S) *vs* B(S). **C** The most enriched GO terms in the top 50 DEGs with the highest fold change in comparison A(S) *vs* C(S). **D** The most enriched GO terms of DEGs in blue module. A: wild type (Col-0); B: *35S::bHLH*-overexpressing transgenic Arabidopsis; C: *P*_*bHLH*_*::bHLH*-overexpressing transgenic *Arabidopsis*; (S): salt treatment.

**Additional file 6: Fig. S6.** Analyses of DEGs in *CgbHLH001* transgenic plants under salt stress. **A** Numbers of DEGs in different comparisons. **B**, **C** Venn analysis of upregulated or downregulated genes in transgenic plants under salt stress. **D**, **E** Go enrichment of upregulated or downregulated genes in transgenic plants under salt stress. In **C**, gene ratio represents the percentage of selected genes, the circle size represents gene numbers, the larger the circle, the more the gene numbers. The color of circle represents the *p* value, the darker the color, the smaller the *p* value, with higher significant difference. The left red font represents abiotic stress related GO terms in biological process.

**Additional file 7: Fig. S7.** Statistical analysis of DE TFs and PKs in different comparisons. A: wild type (Col-0); B: *35S::bHLH*-overexpressing transgenic Arabidopsis; C: *P*_*bHLH*_*::bHLH*-overexpressing transgenic *Arabidopsis*; (C): normal condition; (S): salt treatment.

**Additional file 8: Table S1.** Analysis on *cis*-acting regulatory elements of the promoter of *CgbHLH001* gene.

**Additional file 9: Table S2.** Overview of RNA-seq data.

**Additional file 10: Table S3.** List of top 30 DEGs with the highest fold change of expression level under salt stress.

**Additional file 11: Table S4.** List of DE TFs after salt stress treatment.

**Additional file 12: Table S5.** Selection of putative salt stress-associated DEGs in transgenic Arabidopsis.

**Additional file 13: Table S6.** Primers used in the present study.

**Additional file 14: Supporting Fig. 1.** Original images of gels and blots. **A**: PCR identification of *35S::bHLH*-overexpressing transgenic Arabidopsis. **B**: PCR identification of *P*_*bHLH*_*::bHLH*-overexpressing transgenic Arabidopsis. **C**: RT-PCR identification of transgenic Arabidopsis lines. **D**: Detection of CgbHLH001 expression in C. *glaucum* under different stress treatments. **E**-**H**: Detection of CgbHLH001 expression in transgenic Arabidopsis under normal condition (**E**), 200 mM NaCl (**F**), 300 mM Mannitol (**G**) and 4°C (**H**) treatments. Tubulin acted as the internal reference.

## Data Availability

The sequence information of *CgbHLH001* promoter is available in the NCBI GenBank (https://www.ncbi.nih.gov/genbank/) under accession number of MW544164. All the raw data from the RNA-seq are available in the Sequencing Read Archive (SRA) of NCBI (PRJNA856615).
