# Supplementary material for: Promoter activity and transcriptome analyses decipher functions of CgbHLH001 gene (Chenopodium glaucum L.) in response to abiotic stress
Source: BMC Plant Biol. 2023 Feb 27;23:116. doi: 10.1186/s12870-023-04128-8 (PMC9969703; doi:10.1186/s12870-023-04128-8)
Supplement: Supplementary file 1 — Additional file 1: Fig. S1. Phenotype performance and gene expression of transgenic Arabidopsis lines overexpressing 35S::bHLH and PbHLH::bHLH in response to drought stress. A Transcriptional expression of CgbHLH001 gene. B Translational expression of CgbHLH001 gene. C-D Phenotypic observation and survival percentage of transgenic Arabidopsis. OE35S1, 2: 35S::bHLH-overexpressing transgenic line 1, 2; OEPb1, 2: PbHLH::bHLH-overexpressing transgenic line 1, 2. Different lowercase letters in a indicate significant difference existing between different transgenic lines. [file 12870_2023_4128_MOESM1_ESM.docx]

**Promoter activity and transcriptome analyses decipher functions of *CgbHLH001* gene (*Chenopodium glaucum* L.) in response to abiotic stress**

**Zixin Zhou^1^**^†^**, Juan Wang^2^**^†^**, Qinghui Yu^2^, Haiyan Lan^1^***

^1^Xinjiang Key Laboratory of Biological Resources and Genetic Engineering, College of Life Science and Technology, Xinjiang University, Urumqi 830017, China;

^2^Institute of Horticulture Crops, Xinjiang Academy of Agricultural Science, Urumqi 830091, China

^†^ These authors contributed equally to this work.

*** Correspondence:** Haiyan Lan: E-mail: [**lanhaiyan@xju.edu.cn**](mailto:lanhaiyan@xju.edu.cn)

Additional file 1


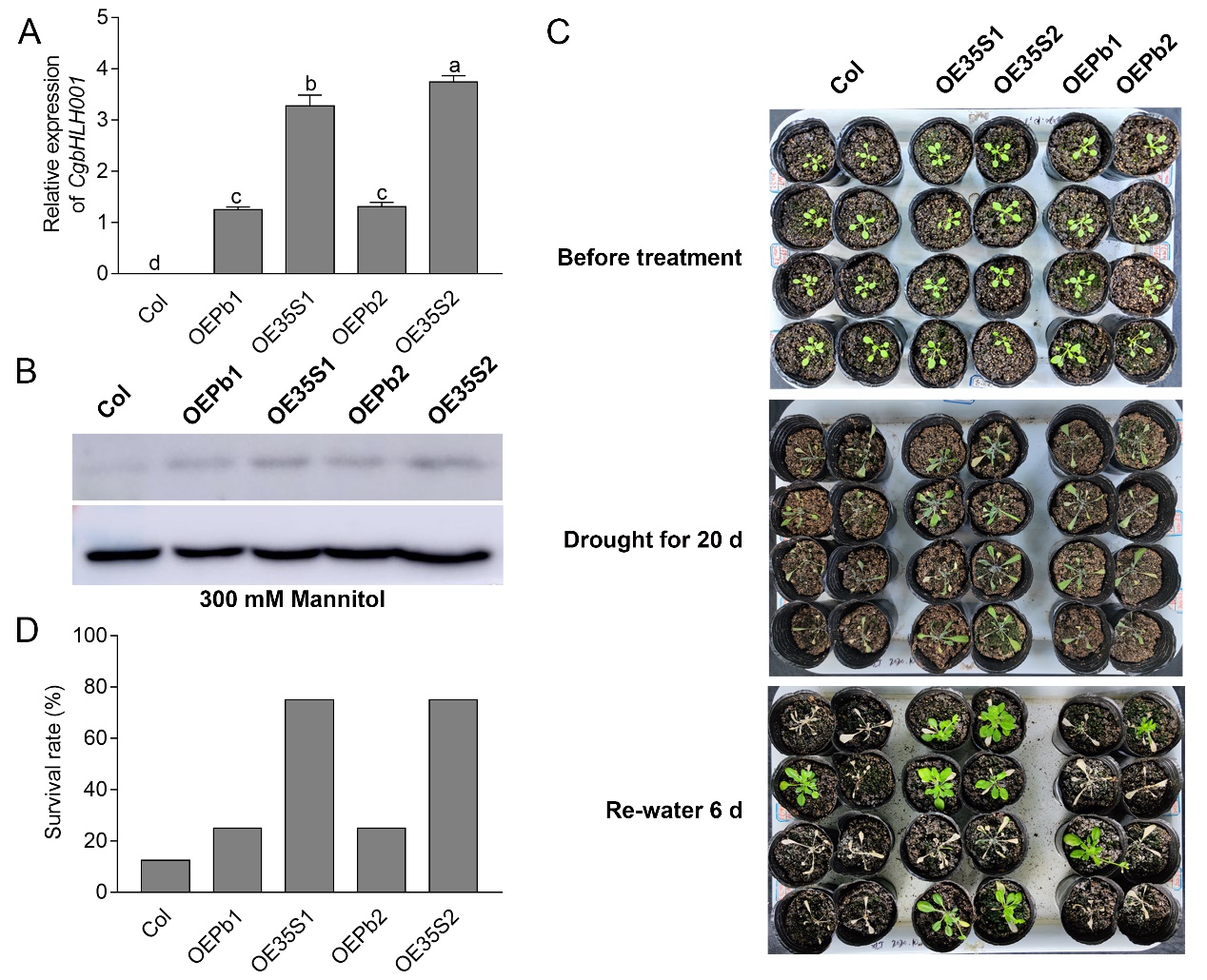


Fig. S1 Phenotype performance and gene expression of transgenic *Arabidopsis* lines overexpressing *35S::bHLH* and *P_bHLH_::bHLH* in response to drought stress. **A** Transcriptional expression of *CgbHLH001* gene. **B** Translational expression of *CgbHLH001* gene. **C**-**D** Phenotypic observation and survival percentage of transgenic *Arabidopsis*. OE35S1, 2: *35S::bHLH*-overexpressing transgenic line 1, 2; OEPb1, 2: *P_bHLH_::bHLH*-overexpressing transgenic line 1, 2. Different lowercase letters in a indicate significant difference existing between different transgenic lines.
