# Supplementary material for: Promoter activity and transcriptome analyses decipher functions of CgbHLH001 gene (Chenopodium glaucum L.) in response to abiotic stress
Source: BMC Plant Biol. 2023 Feb 27;23:116. doi: 10.1186/s12870-023-04128-8 (PMC9969703; doi:10.1186/s12870-023-04128-8)
Supplement: Supplementary file 2 — Additional file 2: Fig. S2. Phenotype performance and gene expression of transgenic Arabidopsis lines overexpressing 35S::bHLH and PbHLH::bHLH in response to 4°C treatment. A Transcriptional expression of CgbHLH001 gene. B Translational expression of CgbHLH001 gene. C-D Phenotypic observation and survival percentage of transgenic Arabidopsis. OE35S1, 2: 35S::bHLH-overexpressing transgenic line 1, 2; OEPb1, 2: PbHLH::bHLH-overexpressing transgenic line 1, 2. Different lowercase letters in A, D indicate significant difference existing between different transgenic lines. [file 12870_2023_4128_MOESM2_ESM.docx]

Additional file 2


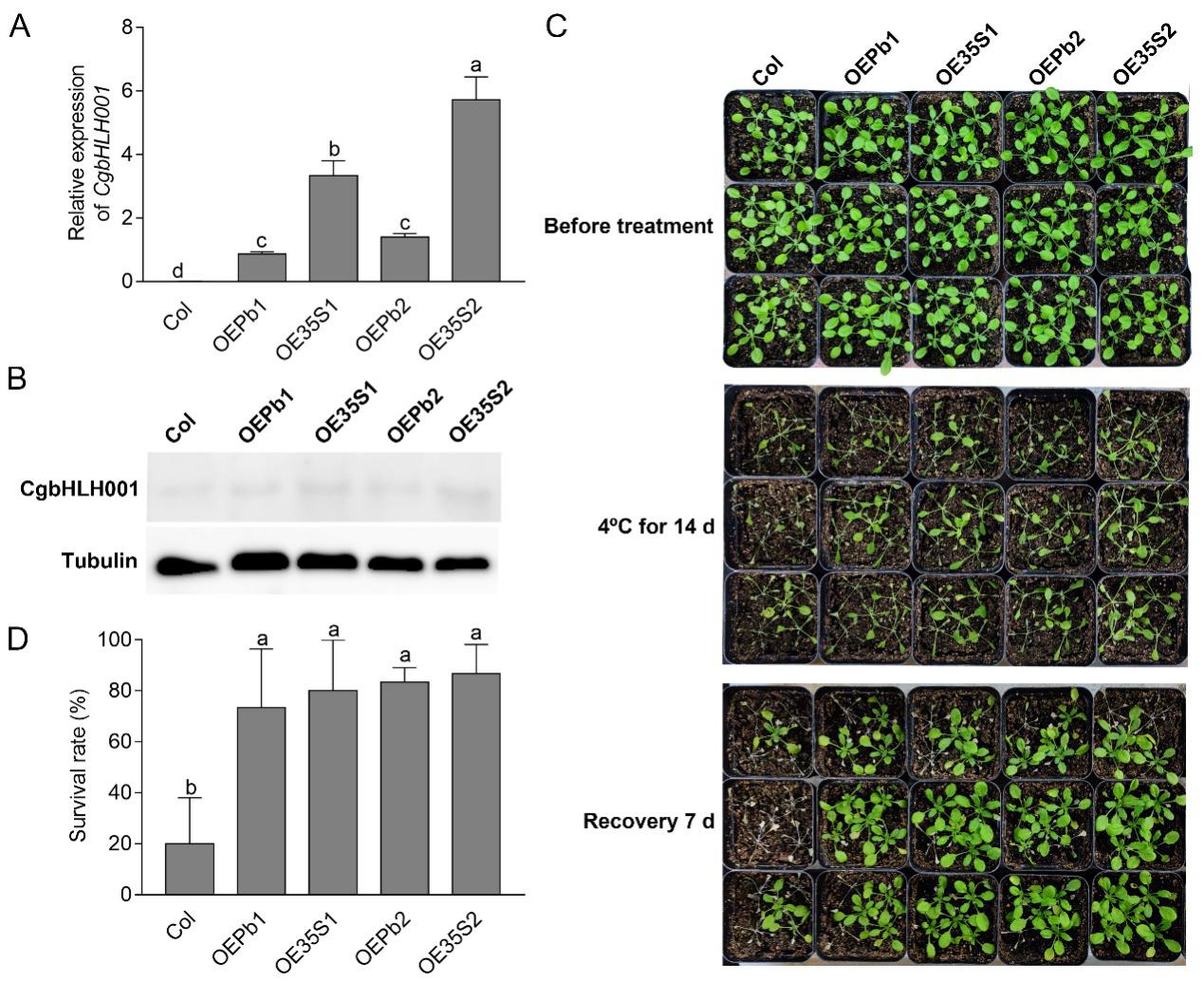


Fig. S2 Phenotype performance and gene expression of transgenic *Arabidopsis* lines overexpressing *35S::bHLH* and *P_bHLH_::bHLH* in response to 4°C treatment. **A** Transcriptional expression of *CgbHLH001* gene. **B** Translational expression of *CgbHLH001* gene. **C**-**D** Phenotypic observation and survival percentage of transgenic *Arabidopsis*. OE35S1, 2: *35S::bHLH*-overexpressing transgenic line 1, 2; OEPb1, 2: *P_bHLH_::bHLH*-overexpressing transgenic line 1, 2. Different lowercase letters in A, D indicate significant difference existing between different transgenic lines.
