# Supplementary material for: Promoter activity and transcriptome analyses decipher functions of CgbHLH001 gene (Chenopodium glaucum L.) in response to abiotic stress
Source: BMC Plant Biol. 2023 Feb 27;23:116. doi: 10.1186/s12870-023-04128-8 (PMC9969703; doi:10.1186/s12870-023-04128-8)
Supplement: Supplementary file 3 — Additional file 3: Fig. S3. The heatmap of correlation analysis (A) and principal component analysis (B) among replicates in the same group and between different groups. A: wild type (Col-0); B: 35S::bHLH-overexpressing transgenic Arabidopsis; C: PbHLH::bHLH-overexpressing transgenic Arabidopsis; (C): normal condition; (S): salt treatment. [file 12870_2023_4128_MOESM3_ESM.docx]

Additional file 3


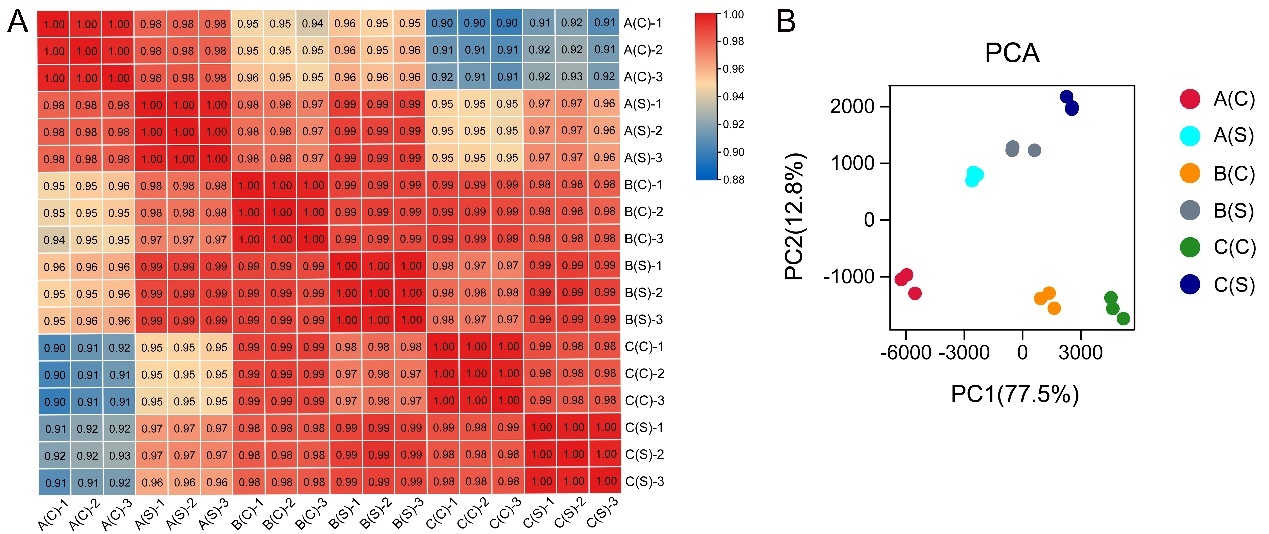


Fig. S3. The heatmap of correlation analysis (**A**) and principal component analysis (**B**) among replicates in the same group and between different groups. A: wild type (Col-0); B: *35S::bHLH*-overexpressing transgenic Arabidopsis; C: *P_bHLH_::bHLH*-overexpressing transgenic Arabidopsis; (C): normal condition; (S): salt treatment.
