# Supplementary material for: Promoter activity and transcriptome analyses decipher functions of CgbHLH001 gene (Chenopodium glaucum L.) in response to abiotic stress
Source: BMC Plant Biol. 2023 Feb 27;23:116. doi: 10.1186/s12870-023-04128-8 (PMC9969703; doi:10.1186/s12870-023-04128-8)
Supplement: Supplementary file 4 — Additional file 4: Fig. S4. The most enriched GO terms in different comparisons. A-B 542 upregulated and 494 downregulated DEGs in A(C) vs C(C); C-D 88 upregulated and 207 downregulated DEGs in A(C) vs B(C); E-F 182 upregulated and 415 downregulated DEGs in the overlap between A(C) vs C(C) and A(C) vs B(C). A: wild type (Col-0); B: 35S::bHLH-overexpressing transgenic Arabidopsis; C: PbHLH::bHLH-overexpressing transgenic Arabidopsis; (C): normal condition. [file 12870_2023_4128_MOESM4_ESM.docx]

Additional file 4


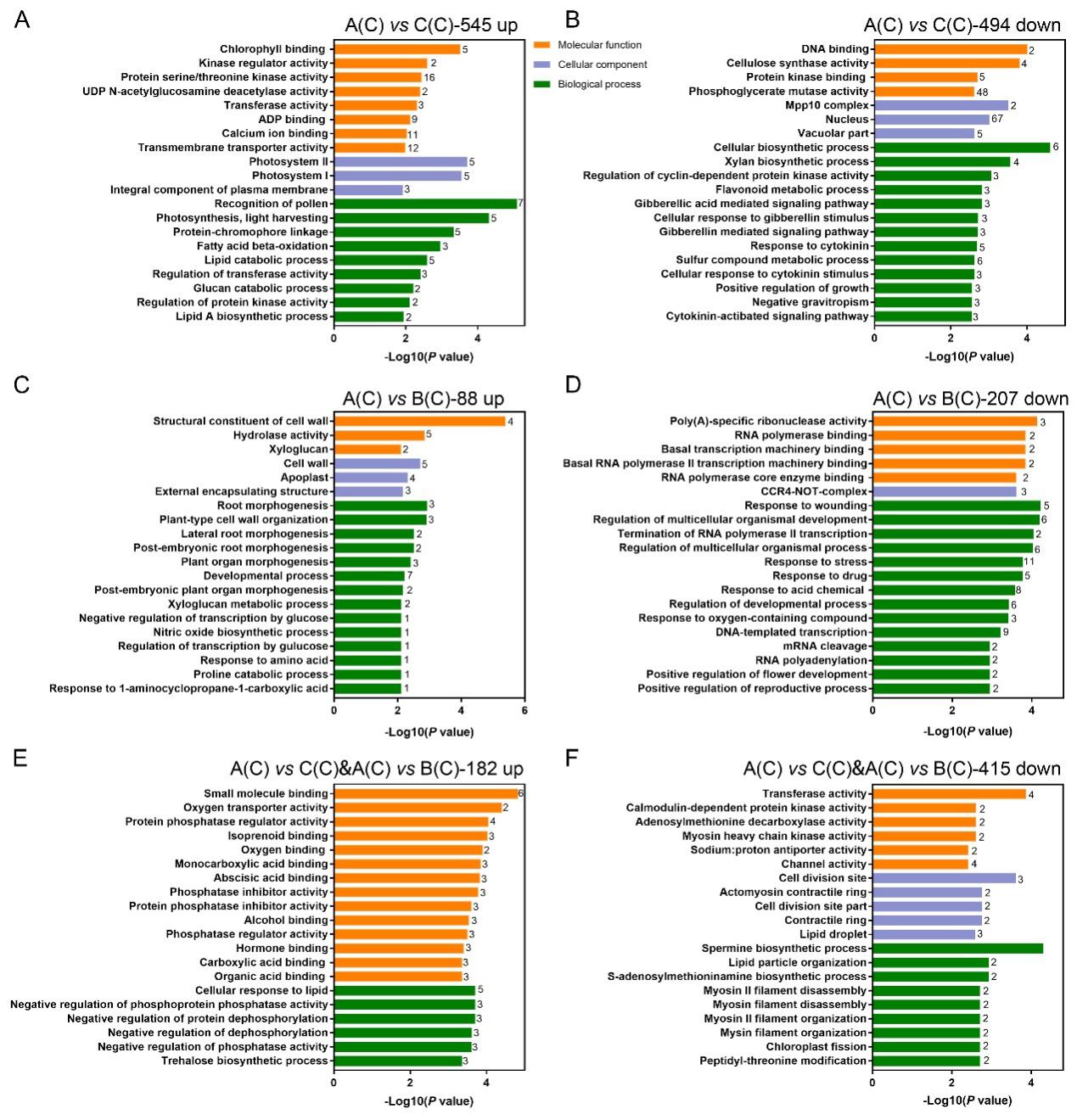


Fig. S4. The most enriched GO terms in different comparisons. **A-B** 542 upregulated and 494 downregulated DEGs in A(C) *vs* C(C); **C-D** 88 upregulated and 207 downregulated DEGs in A(C) *vs* B(C); **E-F** 182 upregulated and 415 downregulated DEGs in the overlap between A(C) *vs* C(C) and A(C) *vs* B(C). A: wild type (Col-0); B: *35S::bHLH*-overexpressing transgenic Arabidopsis; C: *P_bHLH_::bHLH*-overexpressing transgenic Arabidopsis; (C): normal condition.
