# Supplementary material for: Promoter activity and transcriptome analyses decipher functions of CgbHLH001 gene (Chenopodium glaucum L.) in response to abiotic stress
Source: BMC Plant Biol. 2023 Feb 27;23:116. doi: 10.1186/s12870-023-04128-8 (PMC9969703; doi:10.1186/s12870-023-04128-8)
Supplement: Supplementary file 5 — Additional file 5: Fig. S5. The most enriched GO terms in various comparisons. A The most enriched GO terms in the top 50 DEGs with the highest fold change in comparison B(S) vs C(S). B The most enriched GO terms in the top 50 DEGs with the highest fold change in comparison A(S) vs B(S). C The most enriched GO terms in the top 50 DEGs with the highest fold change in comparison A(S) vs C(S). D The most enriched GO terms of DEGs in blue module. A: wild type (Col-0); B: 35S::bHLH-overexpressing transgenic Arabidopsis; C: PbHLH::bHLH-overexpressing transgenic Arabidopsis; (S): salt treatment. [file 12870_2023_4128_MOESM5_ESM.docx]

Additional file 5


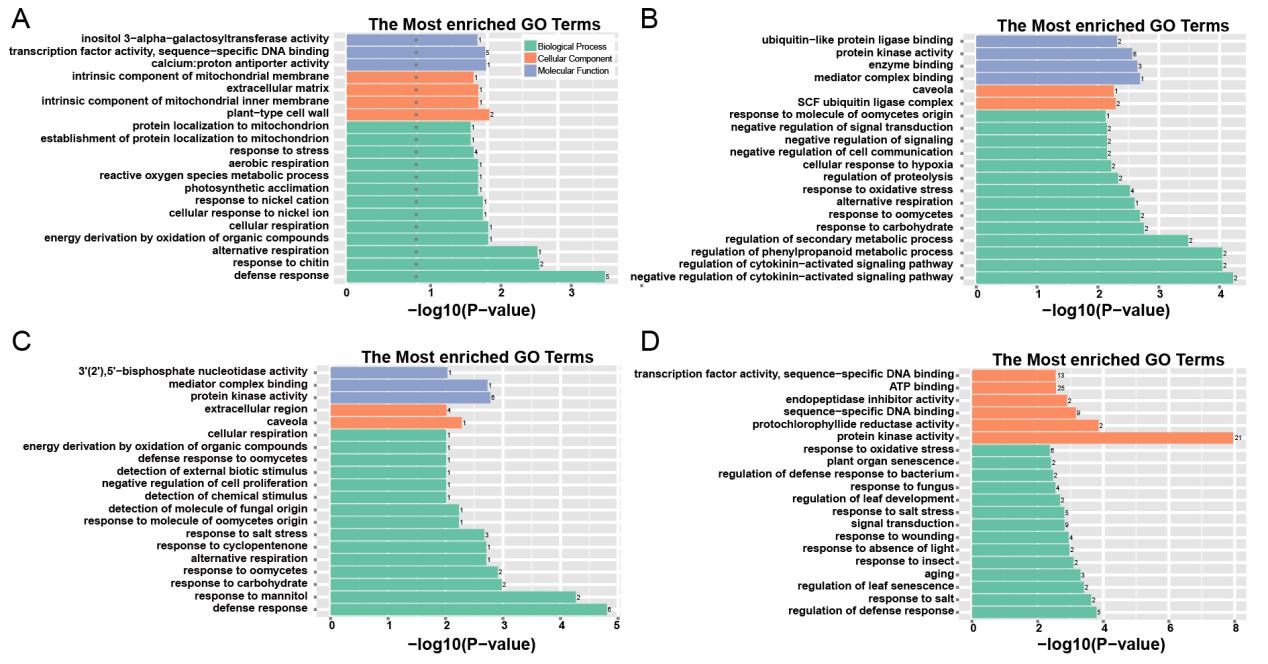


Fig. S5. The most enriched GO terms in various comparisons. **A** The most enriched GO terms in the top 50 DEGs with the highest fold change in comparison B(S) *vs* C(S). **B** The most enriched GO terms in the top 50 DEGs with the highest fold change in comparison A(S) *vs* B(S). **C** The most enriched GO terms in the top 50 DEGs with the highest fold change in comparison A(S) *vs* C(S). **D** The most enriched GO terms of DEGs in blue module. A: wild type (Col-0); B: *35S::bHLH*-overexpressing transgenic Arabidopsis; C: *P_bHLH_::bHLH*-overexpressing transgenic Arabidopsis; (S): salt treatment.
