# Supplementary material for: Promoter activity and transcriptome analyses decipher functions of CgbHLH001 gene (Chenopodium glaucum L.) in response to abiotic stress
Source: BMC Plant Biol. 2023 Feb 27;23:116. doi: 10.1186/s12870-023-04128-8 (PMC9969703; doi:10.1186/s12870-023-04128-8)
Supplement: Supplementary file 6 — Additional file 6: Fig. S6. Analyses of DEGs in CgbHLH001 transgenic plants under salt stress. A Numbers of DEGs in different comparisons. B, C Venn analysis of upregulated or downregulated genes in transgenic plants under salt stress. D, E Go enrichment of upregulated or downregulated genes in transgenic plants under salt stress. In C, gene ratio represents the percentage of selected genes, the circle size represents gene numbers, the larger the circle, the more the gene numbers. The color of circle represents the p value, the darker the color, the smaller the p value, with higher significant difference. The left red font represents abiotic stress related GO terms in biological process. [file 12870_2023_4128_MOESM6_ESM.docx]

Additional file 6


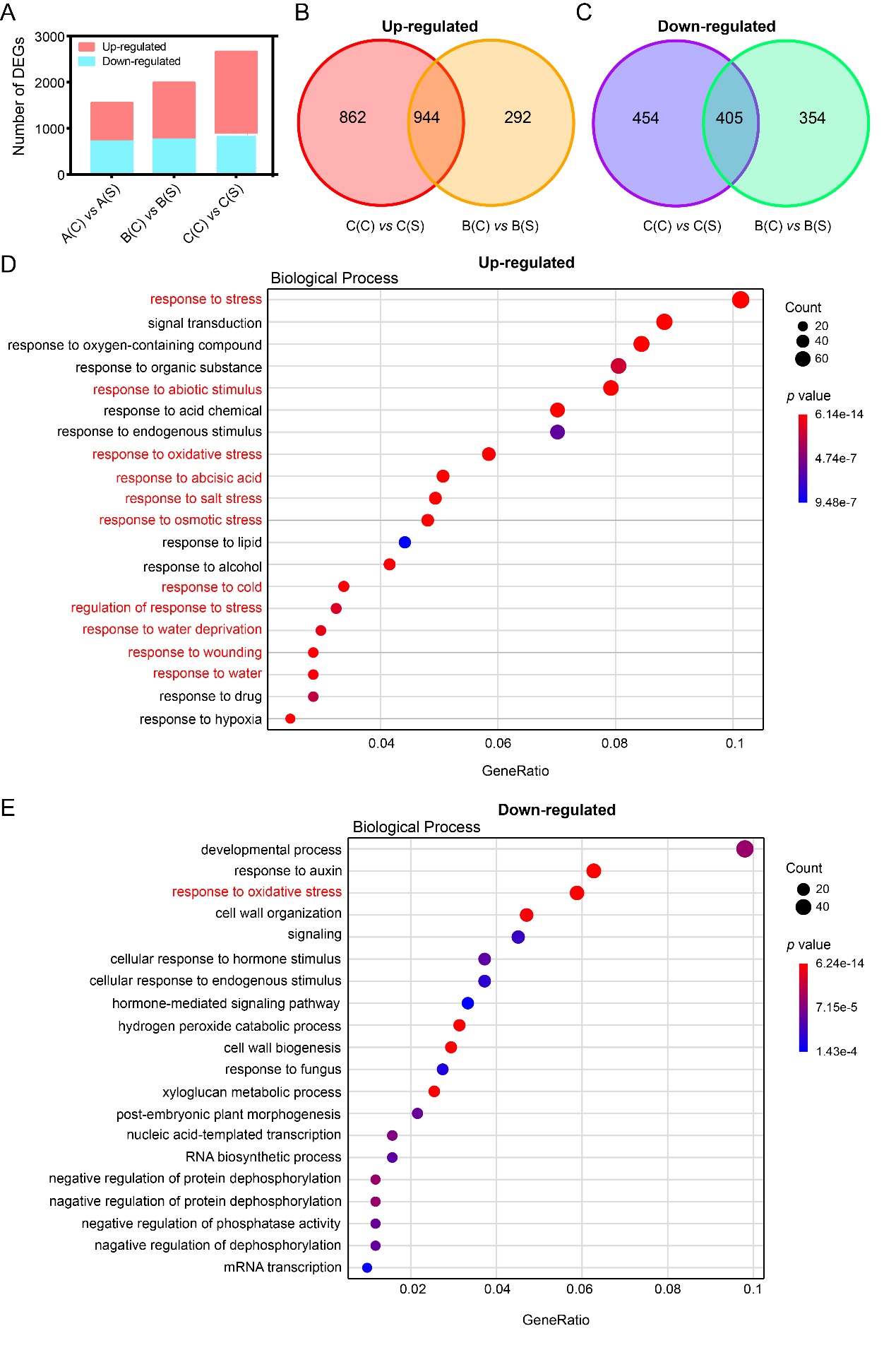


Fig. S6. Analyses of DEGs in *CgbHLH001* transgenic plants under salt stress. **A** Numbers of DEGs in different comparisons. **B, C** Venn analysis of upregulated or downregulated genes in transgenic plants under salt stress. **D, E** Go enrichment of upregulated or downregulated genes in transgenic plants under salt stress. In C, gene ratio represents the percentage of selected genes, the circle size represents gene numbers, the larger the circle, the more the gene numbers. The color of circle represents the *p* value, the darker the color, the smaller the *p* value, with higher significant difference. The left red font represents abiotic stress related GO terms in biological process.
