# Supplementary material for: Promoter activity and transcriptome analyses decipher functions of CgbHLH001 gene (Chenopodium glaucum L.) in response to abiotic stress
Source: BMC Plant Biol. 2023 Feb 27;23:116. doi: 10.1186/s12870-023-04128-8 (PMC9969703; doi:10.1186/s12870-023-04128-8)
Supplement: Supplementary file 8 — Additional file 8: Table S1. Analysis on cis-acting regulatory elements of the promoter of CgbHLH001 gene. [file 12870_2023_4128_MOESM8_ESM.docx]

Additional file 8

Table S1 Analysis on *cis*-acting regulatory elements of the promoter of *CgbHLH001* gene

| *Cis*-element name | Sequence | Function |
| --- | --- | --- |
| 5’UTR Py rich | TTTCTCTCTCT | *Cis*-acting element conferring high transcription levels |
| MYB recognize site | CCGTTG | MYB recognize site |
| MBS/Myb | CAACTG | MYB binding site, response to drought |
| CARE | CAACTCCC | Common *cis*-acting element in the regions of promoter and enhancer |
| TATA-box | TATA | Core promoter element |
| TGACG motif/as-1 | TGACG | Response MeJA |
| ABRE | ACGTG | *Cis*-acting element involved in ABA responsiveness |
| CAAT | CAAT/CAAAT | *Cis*-acting element of promoter and enhancer |
| WRE3 | CCACCT |  |
| MYC | CATTTG | Dehydration response gene *RD22* binding site |
| ACTCATCCT sequence | ACTCATCCT |  |
| TCA element | TCATCATCAT | Response to SA |
| G-box | TACGTG | Response to light |
| Box4 | TGGTTT | Response to light |
| WUN motif | AAATTACTA | Response to wound |
| W-box | TTGACC | Response to disease, ABA, GA and wound, WRKY binding site |
