# Supplementary material for: Promoter activity and transcriptome analyses decipher functions of CgbHLH001 gene (Chenopodium glaucum L.) in response to abiotic stress
Source: BMC Plant Biol. 2023 Feb 27;23:116. doi: 10.1186/s12870-023-04128-8 (PMC9969703; doi:10.1186/s12870-023-04128-8)
Supplement: Supplementary file 9 — Additional file 9: Table S2. Overview of RNA-seq data. [file 12870_2023_4128_MOESM9_ESM.docx]

Additional file 9

Table S2. Overview of RNA-seq data

| Samples | Clean reads | %≥Q30 | Total Reads | mapped Reads |
| --- | --- | --- | --- | --- |
| A0-1 | 37,601,818 | 93.63% | 75203636 (100%) | 71059073 (94.49%) |
| A0-2 | 34,910,074 | 92.46% | 69820148 (100%) | 65016963 (93.12%) |
| A0-3 | 31,724,192 | 94.75% | 63448384 (100%) | 60723621 (95.71%) |
| A1-1 | 23,131,541 | 93.45% | 46263082 (100%) | 43729181 (94.52%) |
| A1-2 | 38,027,179 | 94.58% | 76054358 (100%) | 72587029 (95.44%) |
| A1-3 | 37,816,307 | 94.66% | 75632614 (100%) | 72346261 (95.65%) |
| B0-1 | 31,516,312 | 91.86% | 63032624 (100%) | 58719841 (93.16%) |
| B0-2 | 35,886,307 | 93.88% | 71772614 (100%) | 68034133 (94.79%) |
| B0-3 | 28,552,060 | 91.02% | 57104120 (100%) | 52892355 (92.62%) |
| B1-1 | 32,306,942 | 92.98% | 64613884 (100%) | 61230095 (94.76%) |
| B1-2 | 37,936,379 | 93.92% | 75872758 (100%) | 71969277 (94.86%) |
| B1-3 | 37,328,882 | 93.40% | 74657764 (100%) | 70767932 (94.79%) |
| C0-1 | 34,745,470 | 93.20% | 69490940 (100%) | 65728095 (94.59%) |
| C0-2 | 33,225,506 | 93.99% | 66451012 (100%) | 63207506 (95.12%) |
| C0-3 | 29,529,818 | 91.59% | 59059636 (100%) | 54562418 (92.39%) |
| C1-1 | 35,462,846 | 93.57% | 70925692 (100%) | 67626457 (95.35%) |
| C1-2 | 33,330,430 | 93.31% | 66660860 (100%) | 63386533 (95.09%) |
| C1-3 | 31,297,795 | 93.60% | 62595590 (100%) | 59305935 (94.74%) |
