# Supplementary material for: Promoter activity and transcriptome analyses decipher functions of CgbHLH001 gene (Chenopodium glaucum L.) in response to abiotic stress
Source: BMC Plant Biol. 2023 Feb 27;23:116. doi: 10.1186/s12870-023-04128-8 (PMC9969703; doi:10.1186/s12870-023-04128-8)
Supplement: Supplementary file 10 — Additional file 10: Table S3. List of top 30 DEGs with the highest fold change of expression level under salt stress. [file 12870_2023_4128_MOESM10_ESM.docx]

Additional file 10

Table S3. List of top 30 DEGs with the highest fold change of expression level under salt stress

| Unique DEGs in B(C) *vs* B(S) | | | Common DEGs in two comparisons | | | Unique DEGs in C(C) *vs* C(S) | | |
| --- | --- | --- | --- | --- | --- | --- | --- | --- |
| Gene name | Log2FC | NR_annotation | Gene name | Log2FC | NR_annotation | Gene name | log2FC | NR_annotation |
| FLOT2 | 1.765644 | SPFH/Band 7/PHB domain-containing membrane-associated protein family | SAG113 | 4.881802 | PP2C protein (Clade A protein phosphatases type 2C) | AT5G50360 | 3.671009 | von willebrand factor A domain protein |
| HSFA2 | 1.695142 | heat shock transcription factor A2 | TSPO | 4.687864 | TSPO (outer membrane tryptophan-rich sensory protein)-like protein | LEA7 | 3.552495 | Late embryogenesis abundant protein (LEA) family protein |
| WAK3 | 1.61016 | wall associated kinase 3 | ERF053 | 4.673812 | Ethylene-responsive transcription factor | BGLU24 | 3.419074 | beta glucosidase 24 |
| AT5G45000 | 1.548268 | Disease resistance protein (TIR-NBS-LRR class) family | ERF054 | 4.137428 | Integrase-type DNA-binding superfamily protein | HSFA6B | 2.971382 | heat shock transcription factor A6B |
| AT1G13470 | 1.492753 | hypothetical protein (DUF1262) | AT4G33467 | 3.949668 | unknown, partial | GOLS4 | 2.582499 | galactinol synthase 4 |
| CAD8 | 1.429759 | cinnamyl alcohol dehydrogenase 8 | RD29A | 3.935952 | low-temperature-responsive protein 78 (LTI78) / desiccation-responsive protein 29A (RD29A) | XTH20 | 2.265691 | xyloglucan endotransglucosylase/hydrolase 20 |
| AT5G55460 | 1.416957 | Bifunctional inhibitor/lipid-transfer protein/seed storage 2S albumin superfamily protein | LTI65 | 3.933966 | CAP160 protein | AT5G59490 | 2.259036 | Haloacid dehalogenase-like hydrolase (HAD) superfamily protein |
| EDS1B | 1.353832 | alpha/beta-Hydrolases superfamily protein | NAC019 | 3.910468 | NAC domain containing protein 19 | BHLH92 | 2.170091 | basic helix-loop-helix (bHLH) DNA-binding superfamily protein |
| HIPP02 | 1.285223 | Heavy metal transport/detoxification superfamily protein | PUB19 | 3.896602 | ARM repeat superfamily protein | AtRLP28 | 2.157514 | receptor like protein 28 |
| AT5G54585 | 1.27709 | hypothetical protein AT5G54585 | AT5G03210 | 3.888513 | E3 ubiquitin-protein ligase | MYB49 | 2.120457 | myb domain protein 49 |
| LSU3 | 1.241319 | response to low sulfur 3 | AIP1 | 3.62848 | highly ABA-induced PP2C protein 2 | NAMT1 | 2.073929 | S-adenosyl-L-methionine-dependent methyltransferases superfamily protein |
| CRK37 | 1.240528 | cysteine-rich RLK (RECEPTOR-like protein kinase) 37 | AT1G11210 | 3.586391 | cotton fiber protein, putative (DUF761) | OXI1 | 2.061763 | AGC (cAMP-dependent, cGMP-dependent and protein kinase C) kinase family protein |
| AT3G48640 | 1.24032 | transmembrane protein | SIS | 3.565331 | E3 ubiquitin-protein ligase RLIM-like protein | LOX4 | 2.010659 | PLAT/LH2 domain-containing lipoxygenase family protein |
| AT1G51890 | 1.23072 | Leucine-rich repeat protein kinase family protein | EDL3 | 3.420032 | EID1-like 3 | AT4G20835 | 1.985499 | hypothetical protein AT4G20835 |
| AT3G57460 | 1.216134 | catalytic/ metal ion binding / metalloendopeptidase/ zinc ion binding protein | PHO1-H10 | 3.406117 | EXS (ERD1/XPR1/SYG1) family protein | AT5G40000 | 1.932838 | P-loop containing nucleoside triphosphate hydrolases superfamily protein |
| AT2G15960 | 1.205267 | stress-induced protein | AT4G33905 | 3.375676 | Peroxisomal membrane 22 kDa (Mpv17/PMP22) family protein | AT1G64590 | 1.903 | NAD(P)-binding Rossmann-fold superfamily protein |
| AT1G57630 | 1.198529 | Toll-Interleukin-Resistance (TIR) domain family protein | AT1G51090 | 3.374312 | Heavy metal transport/detoxification superfamily protein | LECRK52 | 1.869383 | Concanavalin A-like lectin protein kinase family protein |
| FOX1 | 1.190816 | FAD-binding Berberine family protein | AFP1 | 3.361395 | ABI five binding protein | UGT79B9 | 1.773683 | UDP-Glycosyltransferase superfamily protein |
| AT1G09500 | 1.186119 | NAD(P)-binding Rossmann-fold superfamily protein | ERD7 | 3.256097 | Senescence/dehydration-associated protein-like protein | AT3G03170 | 1.725337 | hypothetical protein AT3G03170 |
| LHT7 | 1.182639 | LYS/HIS transporter 7 | NAC055 | 3.191285 | NAC domain containing protein 3 | AT3G46650 | 1.706315 | UDP-Glycosyltransferase superfamily protein |
| CRK4 | 1.143935 | cysteine-rich RLK (RECEPTOR-like protein kinase) 4 | LBD1 | 3.183012 | LOB domain-containing protein 1 | GLR1.3 | 1.693284 | glutamate receptor 1.3 |
| AT5G59670 | 1.126504 | Leucine-rich repeat protein kinase family protein | New Gene 209 | 3.181889 | -- | AT5G23830 | 1.683692 | MD-2-related lipid recognition domain-containing protein |
| DMR6 | 1.118653 | 2-oxoglutarate (2OG) and Fe(II)-dependent oxygenase superfamily protein | PXG3 | 3.179017 | Caleosin-related family protein | BGLU22 | 1.662682 | Glycosyl hydrolase superfamily protein |
| AT1G02360 | 1.105288 | Chitinase family protein | LEA46 | 3.167883 | Late Embryogenesis Abundant 4-5 | RBOHC | 1.659042 | NADPH/respiratory burst oxidase protein D |
| CNGC3 | 1.094458 | cyclic nucleotide gated channel 3 | AT5G17460 | 3.12919 | glutamyl-tRNA (Gln) amidotransferase subunit C | FH12 | 1.647059 | Actin-binding FH2 (formin homology 2) family protein |
| BGL2 | 1.092505 | beta-1,3-glucanase 2 | AFP3 | 3.127636 | ABI five binding protein 3 | PER15 | 1.641349 | Peroxidase superfamily protein |
| PMAT1 | 1.077582 | HXXXD-type acyl-transferase family protein | AT1G49450 | 3.121055 | Transducin/WD40 repeat-like superfamily protein | GLP9 | 1.63578 | germin-like protein 9 |
| WAK1 | 1.062359 | cell wall-associated kinase | GOLS2 | 3.112796 | galactinol synthase 2 | FRO2 | 1.634098 | ferric reduction oxidase 2 |
| CYP81D11 | 1.044599 | Cytochrome P450 superfamily protein | AT3G27250 | 2.996194 | hypothetical protein AT3G27250 | AT4G12410 | 1.631739 | SAUR-like auxin-responsive protein family |
| AT4G21500 | 1.042027 | transmembrane protein | NAC032 | 2.994256 | NAC domain containing protein 32 | HEC1 | 1.602983 | basic helix-loop-helix (bHLH) DNA-binding superfamily protein |
