# Supplementary material for: Promoter activity and transcriptome analyses decipher functions of CgbHLH001 gene (Chenopodium glaucum L.) in response to abiotic stress
Source: BMC Plant Biol. 2023 Feb 27;23:116. doi: 10.1186/s12870-023-04128-8 (PMC9969703; doi:10.1186/s12870-023-04128-8)
Supplement: Supplementary file 11 — Additional file 11: Table S4. List of DE TFs after salt stress treatment. [file 12870_2023_4128_MOESM11_ESM.docx]

Additional file 11

Table S4. List of DE TFs after salt stress treatment

| TF family | B(C) *vs* B(S) | | C(C) *vs* C(S) | |
| --- | --- | --- | --- | --- |
|  | Up | Down | Up | Down |
| AP2/ERF-ERF | 28 | 13 | 40 | 10 |
| C2C2-GATA | 0 | 5 | 0 | 5 |
| C2H2 | 4 | 5 | 10 | 9 |
| MYB-related | 8 | 5 | 6 | 8 |
| bHLH | 7 | 5 | 17 | 9 |
| bZIP | 7 | 5 | 12 | 4 |
| NAC | 21 | 4 | 25 | 3 |
| HSF | 5 | 3 | 6 | 0 |
| MYB | 8 | 3 | 17 | 3 |
| TCP | 0 | 3 | 0 | 3 |
| WRKY | 11 | 3 | 18 | 3 |
| C3H | 3 | 0 | 6 | 0 |
| B3 | 0 | 2 | 0 | 0 |
