# Supplementary material for: Promoter activity and transcriptome analyses decipher functions of CgbHLH001 gene (Chenopodium glaucum L.) in response to abiotic stress
Source: BMC Plant Biol. 2023 Feb 27;23:116. doi: 10.1186/s12870-023-04128-8 (PMC9969703; doi:10.1186/s12870-023-04128-8)
Supplement: Supplementary file 12 — Additional file 12: Table S5. Selection of putative salt stress-associated DEGs in transgenic Arabidopsis. [file 12870_2023_4128_MOESM12_ESM.docx]

Additional file 12

Table S5. Selection of putative salt stress-associated DEGs in transgenic Arabidopsis

| **Gene name** | **Gene ID** | **Product encode** |
| --- | --- | --- |
| **TFs (Proteins)** |  |  |
| ZAT8 | AT3G46080 | C2H2-type zinc finger family protein of *A. thaliana* |
| LTI65 | AT5G52300 | low-temperature-induced 65 kDa protein |
| ABF1 | AT1G49720 | ABRE binding factor |
| GSTF6 | AT1G02930 | glutathione S-transferase 6 |
| PXG3 | AT2G33380 | peroxygenase 3 |
| RLP23 | AT2G32680 | receptor like protein |
| NAC035 | AT2G02450 | NAC domain containing protein 35 of *A. thaliana* |
| **Protein kinases** |  |  |
| MPK11 | AT1G01560 | mitogen-activated protein kinase 11 |
| MPK3 | AT3G45640 | mitogen-activated protein kinase 3 |
| MEK1 | AT4G26070 | MAP kinase/ ERK kinase 1 |
| CAMTA3 | AT2G22300 | calmodulin-binding transcription activator 3 |
| SnRK2 | AT5G66880 | serine/threonine-protein kinase |
| CPK27 | AT4G04700 | calcium-dependent protein kinase 27 |
| **Hormone related genes** | |  |
| AUX/IAA | AT1G04240 | auxin-responsive protein IAA3 |
| PYL4 | AT2G38310 | PYR1-like 4 |
| GH3 | AT5G13320 | auxin-responsive GH3 family protein |
| BRI1 | AT1G35710 | brassinosteroid insensitive 1 |
| **Stress related genes** | |  |
| SAG21 | AT4G02380 | senescence-associated gene 21 |
| ERD7 | AT2G17840 | early responsive to dehydration 7 |
| P5CSB | AT3G55610 | delta 1-pyrroline-5-carboxylate synthase 2 |
| RD29A | AT5G52310 | desiccation-responsive protein 29A |
| LEA2 | AT1G02820 | late embryogenesis abundant protein |
| **Ion transporters** | |  |
| HKT1 | AT4G10310 | high-affinity K^+^ transporter 1 |
| SOS1 | AT2G01980 | salt overly sensitive 1 |
| SOS2 | AT5G35410 | salt overly sensitive2 |
| SOS3 | AT5G24270 | salt overly sensitive3 |
| CLC-B | AT3G27170 | chloride channel protein CLC-b |
| NHX3 | AT5G55470 | Na^+^/H^+^ antiporter 3 |
