# Supplementary material for: Promoter activity and transcriptome analyses decipher functions of CgbHLH001 gene (Chenopodium glaucum L.) in response to abiotic stress
Source: BMC Plant Biol. 2023 Feb 27;23:116. doi: 10.1186/s12870-023-04128-8 (PMC9969703; doi:10.1186/s12870-023-04128-8)
Supplement: Supplementary file 13 — Additional file 13: Table S6. Primers used in the present study. [file 12870_2023_4128_MOESM13_ESM.docx]

Additional file 13

Table S6 Primers used in the present study

| Gene name | Primer sequence（5’-3’） | |
| --- | --- | --- |
|  | Forward | Reverse |
| *Pro_CgbHLH001_-*SP1(walking) | TTCAACGGAGAAGGGAGGAGAGAG | |
| *Pro_CgbHLH001_-*SP2(walking) | GTTTGTTCTCGGGAGTTG | |
| *Pro_CgbHLH001_-*SP3(walking) | TCCACTTTATCGACGGTG | |
| pCAMBIA1300-*Pro_CgbHLH001_-FL* | CCGGAATTCTGACCTTCACTTAACTTG | GACGTCGACAACACCTTCAAAAATCTC |
| pCAMBIA1300-*Pro_CgbHLH001_-960* | CCGGAATTCTGTAATTAACCTCACCAAG | GACGTCGACAACACCTTCAAAAATCTC |
| pCAMBIA1300-*Pro_CgbHLH001_-521* | CCGGAATTCTCCAAAAATATTAAATGC | GACGTCGACAACACCTTCAAAAATCTC |
| pCAMBIA1300-*Pro_CgbHLH001_-364* | CCGGAATTCTACTACTGTTACAGCGGAT | GACGTCGACAACACCTTCAAAAATCTC |
| pCAMBIA1300-*Pro_CgbHLH001_-1148* | CCGGAATTCTGACCTTCACTTAACTTG | GACGTCGACCAGTGGGTAGTCATACATCTAG |
| qRT-*GUS* | ATCCGGTCAGTGGCAGTGAAGG | CAGCGTAAGGGTAATGCGAG |
| qRT-*NtActin* | CTATTCTCCGCTTTGGACTTGGCA | ACCTGCTGGAAGGTGCTGAGGGAA |
| qRT-*AtActin* | GTCTGGATTGGAGGGTC | TGAGAAATGGTCGGAAA |
| *CgbHLH001* (qRT-PCR) | TCATGTTCGAGCGAGGAGAGG | CGGGGACAAGATCTTGGAGTATTC |
| *CgGAPDH* (qRT-PCR) | GTTTTCACTGACAAGGACAAGGCTGCTG | GGTGGTACAACTGGCATTGGAGAC |
| ZAT8 | CCGATATGTGGCGTGGAGTT | CGTCGTCGTCTCCGGTAAAA |
| LTI65 | GGAGTGAAGGAGACGCAACA | CCACCTCCTTTGTAGCCGTT |
| ABF1 | CTTACGTGTTTGGTCGGGGA | AAGCCTGTTTTCGAGCCCTT |
| GSTF6 | CCTTTCATCCTTCGCAACCC | CTGGGTCAAACTCATGCGAC |
| PXG3 | AGGGATTTCGTGACCTTGGTT | ACTCCCATGCTTGGCTTTGT |
| RLP23 | CTGGTATTGTGGCCTGTCGT | CGTCGAGTTATCGCACCAGA |
| MPK11 | ATTTTGGGCTTGCGAGGACT | GGCTCCCGCGTCATTATTTC |
| MPK3 | ACTTCCCAACTTCCCACGTC | TCTGTTGGGGTCAAACGTCA |
| MEK1 | AGCTTGGGACTGGTTTTGCT | AAGGAGGCGGGTTTTCAACA |
| CAMTA3 | TCGCTGGTGTAAGCGTTGAT | AAGGTGTGCTTCCTGATGGG |
| SnRK2 | CGCTCCAGAGGTACTGCTTC | AACGGATACGCTCCAACCAA |
| CPK27 | GAAGCGGACATTTGGAGTGC | ATGGTTCCTCGCTGTAGTCG |
| IAA3 | ACTGAAACATCCCCTCCTCG | CCTTGACCCTCATGCTCAGAT |
| PYL4 | GTGTTGCTCCGCCGTTATTC | GTGGACTTGACGGAGGCTAC |
| GH3 | TACTCGTACACCAGCCCTGA | CGTCCTCACAACCTCGTCTC |
| BRI1 | CCTCGAAGGTCCTATCCCGA | ACCGTGGAATTTGTTGTGGC |
| SAG21 | CGTCTCTCGTGAACTCTCCA | TCTTCATCACAGCCGAAGCA |
| ERD7 | AAGCGACGAAGAAGACGGAG | GCCACCGTCACATCCAAAAC |
| P5CSB | TGTGAACAGCTTGCGGAGTT | ATGAGACTGCTCTGCCCAAC |
| RD29A | GAGCTCCGTTGGGAGGAAAT | GGTCTTCCCTTCGCCAGAAA |
| LEA2 | ATGGCTCGTTCTCTCGCTAA | ATCTAACGCCGTTTTGGCTG |
| CLC-B | TCTGCTTCTCACCACCAACG | AGGAAGAGACCAGACGGTGT |
| NHX3 | TCCGGTGTCATAACCGCATT | ACAGCACCTCGCATTAGACC |
| HKT1 | GTCTCTGCCATCACCGTCTC | GAAGATTTCGCCACCGAGGA |
| SOS1 | TCATCTCCCGCCGCATTATC | CTTGAGCTACGTGGTGGTGT |
| SOS2 | AAGTACGAGGTTGGTCGCAC | GAAGGACTCGCCAACACCTC |
| SOS3 | CGCCGGTCCATGAAAAAGTC | GTCGTTTTTGCGGTCTGCTT |
| NAC035 | CGCCGAAAAGTTGAAGGCAA | CTATCAGCTCCGGTGGCTTT |
