# Supplementary material for: Promoter activity and transcriptome analyses decipher functions of CgbHLH001 gene (Chenopodium glaucum L.) in response to abiotic stress
Source: BMC Plant Biol. 2023 Feb 27;23:116. doi: 10.1186/s12870-023-04128-8 (PMC9969703; doi:10.1186/s12870-023-04128-8)
Supplement: Supplementary file 14 — Additional file 14: Supporting Fig. 1. Original images of gels and blots. A: PCR identification of 35S::bHLH-overexpressing transgenic Arabidopsis. B: PCR identification of PbHLH::bHLH-overexpressing transgenic Arabidopsis. C: RT-PCR identification of transgenic Arabidopsis lines. D: Detection of CgbHLH001 expression in C. glaucum under different stress treatments. E-H: Detection of CgbHLH001 expression in transgenic Arabidopsis under normal condition (E), 200 mM NaCl (F), 300 mM Mannitol (G) and 4°C (H) treatments. Tubulin acted as the internal reference. [file 12870_2023_4128_MOESM14_ESM.docx]

Additional file 14


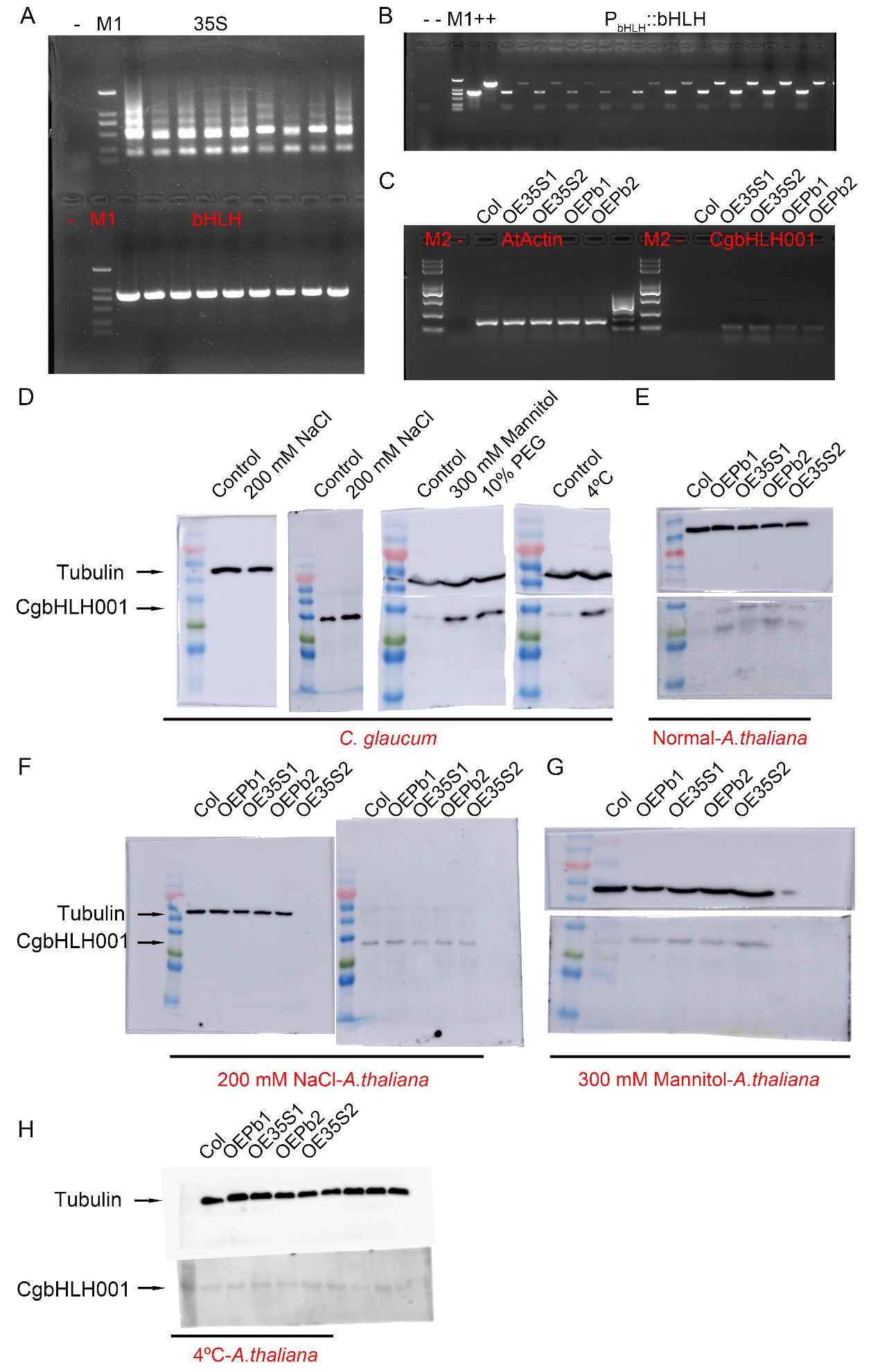


Supporting Fig. 1. Original images of gels and blots. A: PCR identification of *35S::bHLH*-overexpressing transgenic Arabidopsis. B: PCR identification of *P_bHLH_::bHLH*-overexpressing transgenic Arabidopsis. C: RT-PCR identification of transgenic Arabidopsis lines. D: Detection of CgbHLH001 expression in *C. glaucum* under different stress treatments. E-H: Detection of CgbHLH001 expression in transgenic Arabidopsis under normal condition (E), 200 mM NaCl (F), 300 mM Mannitol (G) and 4°C (H) treatments. Tubulin acted as the internal reference.
